# U^4+/5+/6+^ in a Conserved Pseudotetrahedral Imidophosphorane Coordination Sphere

**DOI:** 10.1021/acs.inorgchem.4c04973

**Published:** 2025-01-27

**Authors:** Andrew C. Boggiano, Julie E. Niklas, Maximilian G. Bernbeck, Henry S. La Pierre

**Affiliations:** † School of Chemistry and Biochemistry, Georgia Institute of Technology, Atlanta, Georgia 30332-0400, United States; ‡ Nuclear and Radiological Engineering and Medical Physics Program, School of Mechanical Engineering, Georgia Institute of Technology, Atlanta, Georgia 30332-0400, United States; § Physical Sciences Division, Pacific Northwest National Laboratory, Richland, Washington 99352, United States

## Abstract

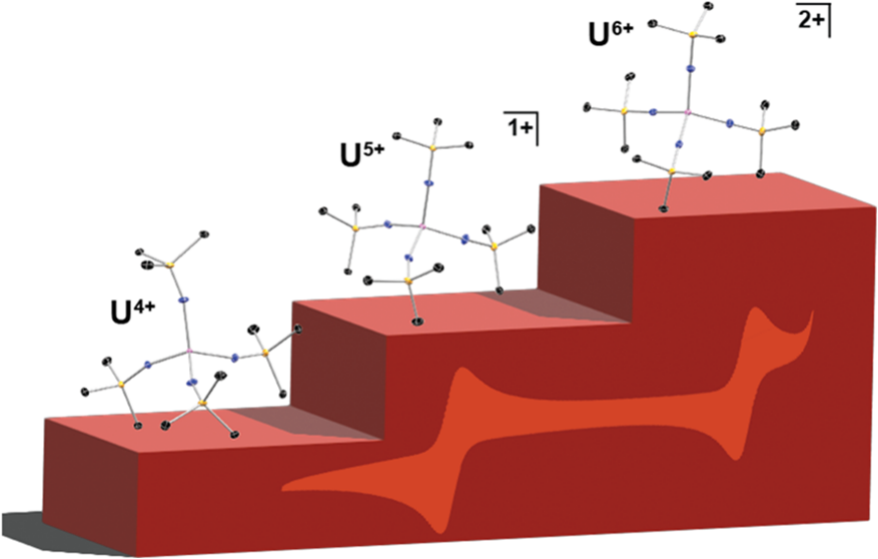

While several ligand systems support uranium across a range of oxidation states, spanning more than two oxidation states in a conserved coordination geometry is uncommon among structurally authenticated complexes. Imidophosphorane ligands significantly stabilize high-valent lanthanide and actinide complexes. Here, we report a series of homoleptic uranium imidophosphorane complexes, spanning the +4, +5 and +6 oxidation states in a four-coordinate pseudotetrahedral ligand field. The +6 oxidation state is accessible using a mild ferrocenium oxidant, yielding a rare example of U^6+^ in a pseudotetrahedral coordination environment. As the formal oxidation state increases, the U–N distances gradually contract, consistent with the Shannon ionic radii of U^4+/5+/6+^. Compared to reported complexes, the short U–N distances observed in the U^6+^ complex are more comparable to dianionic imido ligands than monoanionic amido ligands.

## Abstract 2

The tri*tert*-butyl imidophosphorane ligand stabilizes uranium in its highest oxidation states (+4−+6) within a conserved tetrahomoleptic coordination sphere. Spectroscopic and magnetic studies are consistent with the oxidation state assignments from the single-crystal X-ray diffraction data. The series includes unusual examples of cationic and dicationic, pseudotetrahedral complexes of U^5+^ and U^6+^ in the absence of the ubiquitous *trans*-dioxo moiety of uranyl complexes.

## Introduction

**Figure 1:**
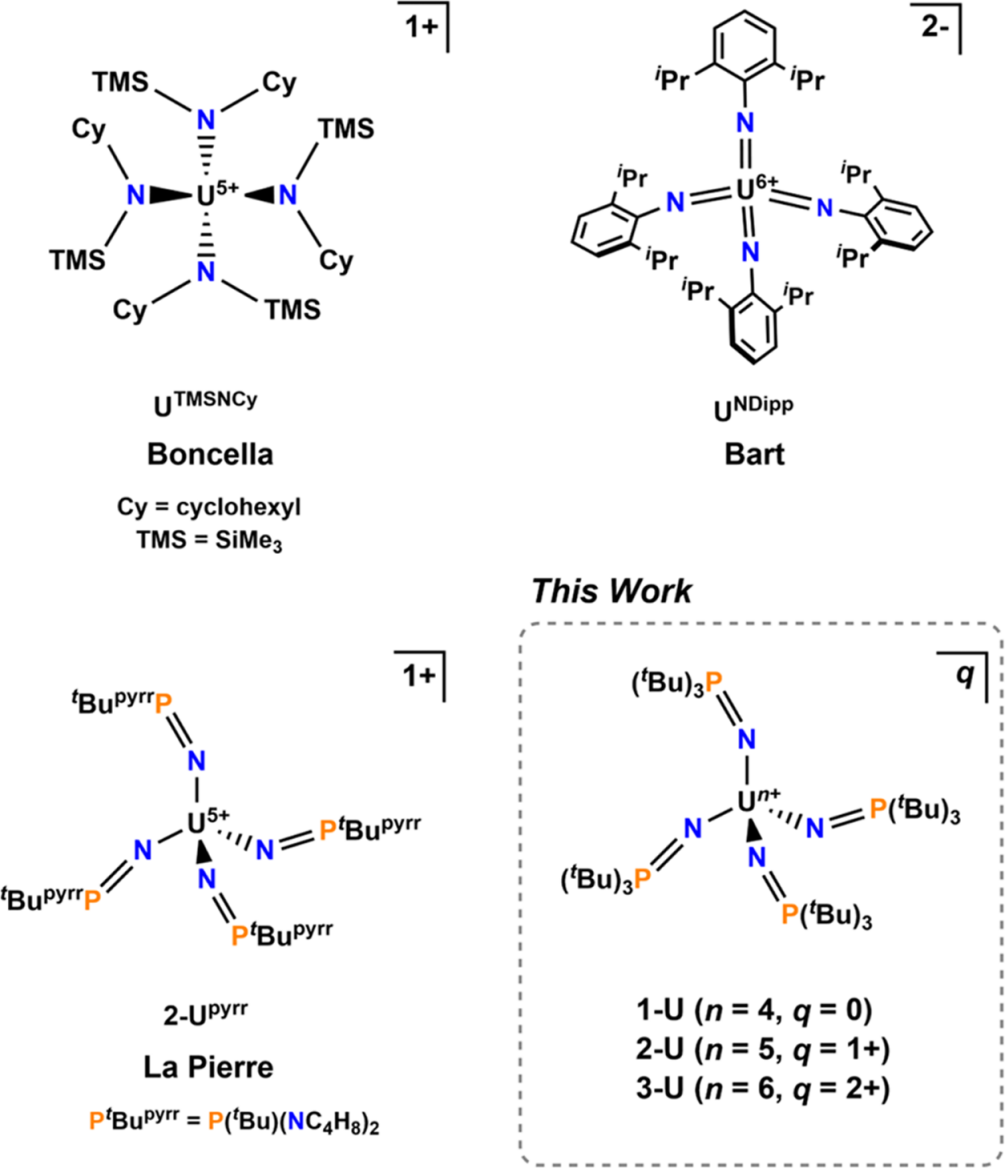
Literature examples of pseudotetrahedral complexes of U^5+^ and U^6+^ and the complexes reported herein.

Complexes of high-valent uranium (>+4 oxidation state) are commonly observed in linear dioxo (uranyl, [O = U^*n*+^=O]^*m*+^, *n* = 5, 6; *m* = n–4) complexes.^1,2^ Aside from uranyl complexes, several U^6+^ mono-oxo or -imido complexes are known, as well as neutral, pseudo-octahedral hexa-halide, alkoxide, alkyl, and amide complexes.^3,4^ Significant progress in the nonaqueous chemistry of uranium has been made in the past few decades, however, accessing U^5+^ and U^6+^ complexes often requires one or more dianionic oxo or imido ligands.^3,5^ Pseudotetrahedral complexes of U^5+^ are rare, however, a couple of examples have been reported (Figure 1).^6,7^ The only pseudotetrahedral complex of U^6+^ was reported by Bart and co-workers in 2017, in an unprecedented dianionic tetrakis-imido complex, [U^6+^(NDipp)_4_][K(2.2.2-cryptand)]_2_ (**U**^**NDipp**^, where Dipp = 2,6-diisopropylphenyl, Figure 1).^8^

Uranium exhibits unique redox behavior, reactivity, and bonding preferences that are distinct from those of the transition metals, lanthanides, and even later actinides.^9^ Metal–ligand multiple bonds are far more common in uranium than in the lanthanide metals; however, the inverse *trans* influence drives coordination geometry preferences distinct from those of the transition metals.^10^ Satisfying or overcoming these preferences to support a range of oxidation states in a conserved geometry is challenging due to the distinct ligand types that support uranium at the extremes of the accessible oxidation states. The characterization of isostructural complexes in a range of oxidation states can provide space to systematically study the fundamental coordination chemistry, bonding preferences, and electronic structure of molecular uranium complexes.

Stabilization of uranium across more than two oxidation states in an isostructural coordination sphere is uncommon. Ligands that support uranium in both high and low formal oxidation states often utilize an anchoring arene ring^11,12^ to support lower oxidation states through δ back-bonding,^13−15^ and the introduction of oxo or imido ligands to stabilize U^5+^ and U^6+^.^11^ The only ligand frameworks reported to support U^4+/5+/6+^ in a structurally authenticated conserved ligand geometry thus far are high-coordinate, heteroleptic, mono-oxo complexes.^11,16,17^ While up to five formal oxidation states have been accessed using an arene anchored amide ligand by Huang and co-workers, the complexes in the series which exhibit a conserved coordination geometry are restricted to the U^4+/5+/6+^ oxo species.^11^ Meyer and co-workers have reported two series of uranium complexes spanning three formal oxidation states in conserved ligand spheres.^16,17^ The first is a series of U^4+/5+/6+^ complexes supported by a tripodal aryloxide and oxido-distorted square antiprismatic coordination geometry.^17^ In 2023, Meyer and co-workers reported an acetylacetonate (acac) ligand framework that supports U^3+/4+/5+^ in a conserved geometry.^16^

Imidophosphorane ligands stabilize high-valent f-element complexes in low-coordinate pseudotetrahedral complexes.^18−22^ While siloxide ligands also stabilize high-valent lanthanide complexes, and are also monoanionic, 1σ, 2π donors, the enhanced stabilizing effect of imidophosphoranes on high-valent lanthanides showcases the distinction between the zwitterionic P–N moiety compared to Si–O.^20,23−30^ Herein, a series of uranium complexes spanning the +4–+6 oxidation states in a conserved coordination geometry is reported, with unusual cationic complexes of U^5+^ and U^6+^ in a pseudotetrahedral ligand sphere.

## Results and Discussion

### Synthesis

**Scheme 1:**
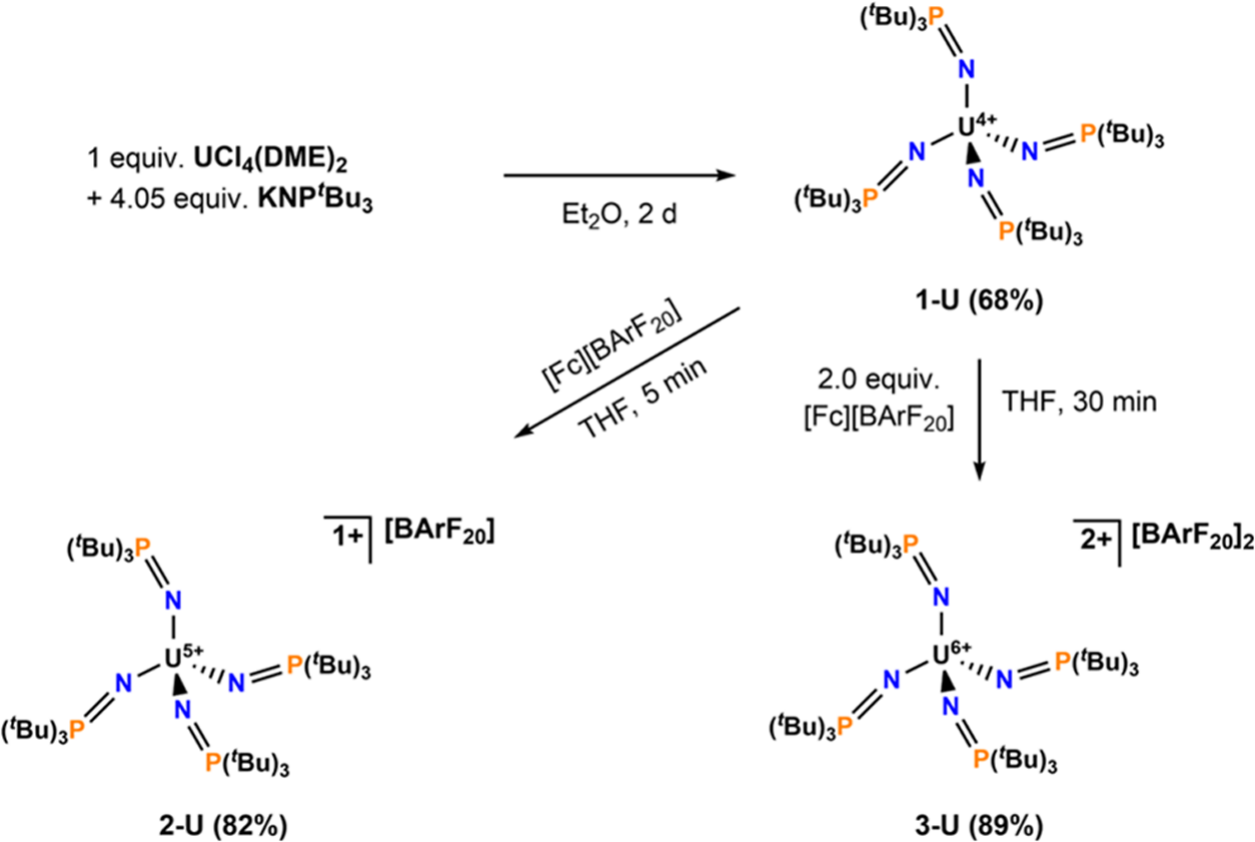
Synthesis of Complexes 1-U, 2-U, and 3-U. [BArF_20_] = tetrakis(pentafluorophenyl) Borate All reactions were performed at room temperature (ca. 25 °C).

The tetravalent uranium complex, [U^4+^(NP^*t*^Bu_3_)_4_] (**1-U**, where ^*t*^Bu = C(CH_3_)_3_), was synthesized by metathesis (Scheme 1) of the potassium salt of the tri-^*t*^Bu imidophosphorane, KNP^*t*^Bu_3_, and UCl_4_(DME)_2_ in diethyl ether (Et_2_O) over 2 d at room temperature (ca. 25 °C). Recrystallization from *n*-pentane yields **1-U** as a pink crystalline solid in 68% yield. Oxidation of **1-U** by one electron with ferrocenium tetrakis(pentafluorophenyl)borate ([Fc][BArF_20_]) in tetrahydrofuran (THF) at room temperature yielded the U^5+^ complex, [U^5+^(NP^*t*^Bu_3_)_4_][BArF_20_] (**2-U**) as a cherry-red crystalline solid in 82% yield after workup (Scheme 1). Addition of two equivalents of [Fc][BArF_20_] to a THF solution of **1-U** yielded the dicationic U^6+^ complex, [U^6+^(NP^*t*^Bu_3_)_4_][BArF_20_]_2_ (**3-U**) as a dark brown/black crystalline solid in 89% yield after crystallization from *ortho*-difluorobenzene layered with Et_2_O/*n*-pentane at −35 °C (Scheme 1). Curiously, reaction between [Ag][BArF_20_] and **1-U** does not proceed at room temperature in THF or Et_2_O, despite the similar or greater redox potential of Ag^+^/Ag^0^ compared to Fc^+^/Fc, depending on the solvent.^31^ The lack of reactivity with [Ag][BArF_20_] despite the apparent thermodynamic accessibility based on electrochemical data was also observed in the oxidation of the Pr^4+^ analogue.^25^

### Structural Analysis

All three complexes were structurally characterized by single-crystal X-ray diffraction (SC-XRD). **1-U** crystallizes in the monoclinic P2_1_/n space group, with one formula unit per asymmetric unit. **2-U** crystallizes in the triclinic P–1 space group, with an asymmetric unit consisting of one formula unit. **2-U** is isotypic to the formally pentavalent praseodymium complex, [Pr^5+^(NP^*t*^Bu_3_)_4_][BArF_20_], with similar unit cell parameters.^25^**3-U** crystallizes in the monoclinic *C*2/*c* space group, with an asymmetric unit of half a formula unit of the uranium complex, and one [BArF_20_]^−^ anion. Due to similar ionic radii and a wider range of accessible oxidation states, the early actinides are aptly suited as structural models of pentavalent lanthanide complexes, with **2-U** establishing an actinide structural parallel to [Pr^5+^(NP^*t*^Bu_3_)_4_][BArF_20_].

**Figure 2:**
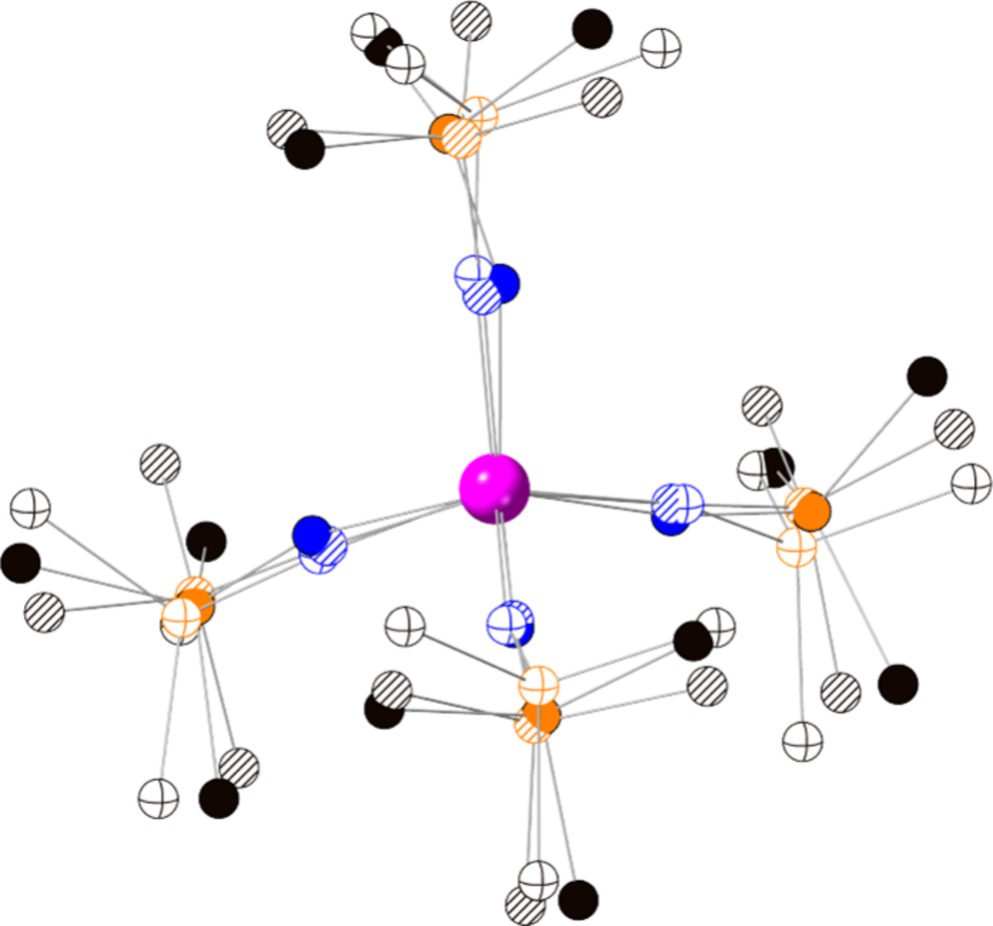
Overlay of **1-U** (hollow fill), **2-U** (solid fill), and **3-U** (hatch pattern) derived from SC-XRD atom coordinates. Legend: Pink, U, Orange, P, Blue, N, Black, C. tert-Butyl groups are truncated and H atoms omitted for clarity. [BArF_20_]^−^ not visually depicted for clarity. Structure of **3-U** is symmetry generated; crystallographic asymmetric unit consists of half formula unit (see SI for details).

All complexes are close to ideal tetrahedral symmetry, with τ_4_ = 0.98 for **1-U** and **3-U**, and 0.96 for **2-U** (Table 1).^33^ A lengthening of the ligand N–P distance is observed upon oxidation, representative of increased donation to the increasingly electropositive uranium center. The mean imidophosphorane N–P distance in **1-U** is 1.556(3) Å, which lengthens by ∼0.07 to 1.625(4) Å in **3-U** (Table 1). The latter value is consistent with the calculated value of 1.618 Å for **3-U**^**pyrr**^.^6^ An overlay of the structures derived from SC-XRD demonstrates the conserved pseudotetrahedral geometry across all three complexes (Figure 2), with relatively small changes in U–N bond lengths as the oxidation state of U changes.

**Table 1:** Averaged Structural Metrics and Calculated Parameters from SC-XRD Data^a^

compound	**1-U**	**2-U**	**3-U**
*d*(U–N) (Å)	2.185(2)	2.107(4)	2.047(12)
*d*(N–P) (Å)	1.556(3)	1.588(1)	1.625(4)
∠(N–U–N) (°)	110(2)	109(3)	110(1)
∠(P–N–U) (°)	165(1)	167(5)	177(1)
τ_4_	0.98	0.96	0.98
∑109.5 (°)^b^	8.5(1)	13.0(2)	7.2(3)

a Numbers in parentheses are estimated standard deviations.

b ∑109.5 is the sum of the deviations from ideal 109.5° of each six angles that make up the pseudo-tetrahedral coordination sphere.

The U–N distances gradually decrease with increase in uranium formal oxidation state, from a mean of 2.185(2) Å in **1-U** to 2.107(4) Å in **2-U**, and 2.047(12) Å in **3-U** (Table 1). Six-coordinate U^4+^ has a Shannon ionic radius of 0.89 Å, which contracts to 0.76 Å for U^5+^, with a modest contraction to 0.73 Å for U^6+^.^32^ The mean of 2.107(4) Å in **2-U** is about 0.09 Å shorter than the mean of 2.189 Å reported by Boncella and co-workers in the pseudotetrahedral U^5+^ complex, [U^5+^(TMSNCy)_4_][BArF_24_] (**U**^**TMSNCy**^, where Cy = cyclohexyl, BArF_24_ = tetrakis(3,5-bis(trifluoromethyl)phenyl)borate), TMS = trimethylsilyl) Figure 1.^7^ The mean U–N distance of 2.047(12) Å in **3-U** agrees with the geometry-optimized mean bond distance of 2.032 Å in the related, computationally modeled^6^ [U^6+^(NP^*t*^Bu(pyrr)_2_)_4_]^2+^ complex, **3-U**^**pyrr**^ (where pyrr = pyrollidinyl, – NC_4_H_8_). In hexavalent **3-U**, the bond distance metrics are similar to the dianionic aryl imido ligands in **U**^**NDipp**^; the U–N distances in **3-U** are actually slightly shorter than the U–N distances in **U**^**NDipp**^, averaging 2.047(12) and 2.062(2) Å, respectively.^8,34^

### Electrochemical Analysis

**Figure 3:**
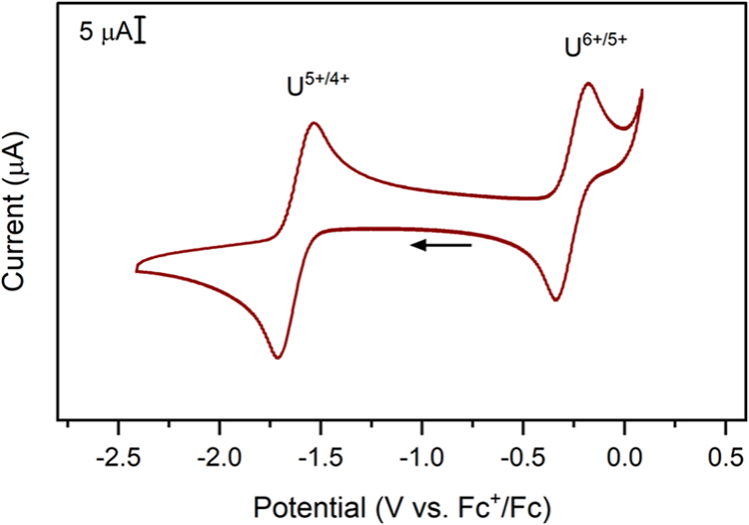
Cyclic voltammogram of 2-U at ∼2.5 mM analyte concentration in 50 mM [^*n*^Bu_4_N][BPh_4_] in THF. Scan rate = 200 mV/s, arrow indicates initial potential and scan direction.

**1-U** and **2-U** were suitable for electrochemical characterization in THF, using 50 mM [^*n*^Bu_4_N][BPh_4_] (where ^*n*^Bu = *n*-butyl, Ph = phenyl) as the supporting electrolyte. The cyclic voltammograms of **1-U** and **2-U** are consistent with each other and feature two quasi-reversible redox events in the electrochemical window (Figures 3, S27, and S30). The U^5+/4+^ couple is observed at −1.62 V vs Fc^+^/Fc for **1-U** and **2-U** at 200 mV/s scan rate, which is similar to the value of −1.57 V. vs Fc^+^/Fc for the previously reported complex, [U^4+^(NP^*t*^Bu(pyrr)_2_)_4_] (**1-U**^**pyrr**^).^6^ Similar to previous studies,^6^ the U^4+/3+^ couple is not observable within the electrochemical window. Dissolution of **3-U** at ∼2.5 mM concentration in 50 mM [^*n*^Bu_4_N][BPh_4_] in THF quickly resulted in the precipitation of a dark brown/black material, similar in appearance to **3-U**. This is likely due to anion exchange with the supporting electrolyte, producing insoluble [U^6+^(NP^*t*^Bu_3_)_4_][BArF_20_]_*x*_[BPh_4_]_2–*x*_ species. Addition of the internal reference, decamethylferrocene (*E*_1/2_ = −0.50 V vs Fc^+^/Fc),^24^ produced a homogeneous cherry-red solution, suggesting chemical reduction to U^5+^. Analysis of this solution displayed a voltammogram similar to the isolated **2-U** (See Figure S34 for comparison).

The U^6+/5+^ couple is measured at −0.27 and −0.26 V vs Fc^+^/Fc at a 200 mV/s scan rate for **1-U** and **2-U**, respectively, which is slightly more positive than the value of −0.32 V vs Fc^+^/Fc reported for **1-U**^**pyrr**^.^6^ The U^6+/5+^ potential of **1-U** and **2-U** is similar to aqueous uranyl (U^6+^O_2_(H_2_O)_*x*_), which has been reported at −0.35 V vs Fc^+^/Fc (converted from −0.17 V vs AgCl/Ag).^2,35^ This potential indicates hexavalent **3-U** is only a mild oxidant,^31^ in comparison to strongly oxidizing U^6+^ complexes, such as UF_6_. The tetrahedral imidophosphorane ligand sphere is therefore similarly stabilizing to the *trans*-dioxo ligands of the uranyl ion. The U^6+/5+^ couples of **1-U** and **2-U** are much more positive than the tris aryl imido complex reported^36^ by Bart and co-workers, with U^6+/5+^ reported at −2.14 vs Fc^+^/Fc.^2^ This suggests that the differences in the formal charges between organic imido and imidophosphorane ligands do substantially modulate the observed redox chemistry. While the zwitterionic character of imidophosphoranes yields a higher stabilization to the metal center compared to siloxide or alkoxide ligands, the dianionic aryl imido ligands shift the redox potential much more negative, rendering U^5+^ a potent reductant in the tris-imido complex reported by Bart & co-workers.^36^ In comparison to other four-coordinate compounds, the potential is comparable to −0.41 and −0.42 V vs Fc^+^/Fc reported for the terminal imido complexes [TMSN=U^5+^(N(TMS)_2_)_3_] and [PhN=U^5+^(N(TMS)_2_)_3_], respectively.^37^

### SQUID Magnetometry

Magnetic susceptibility measurements support the metal oxidation state assignments from the structural and spectroscopic data. Tetravalent **1-U** exhibited a room temperature χ_M_*T* value of 0.95 emu mol^–1^ K (μ_eff_ = 2.76 μ_B_), which is in excellent agreement with the median reported value of 0.97 emu mol^–1^ K (μ_eff_ = 2.79 μ_B_) for complexes of U^4+^.^38^ At 1.8 K, χ_M_*T* of **1-U** approaches zero (0.02 emu mol^–1^ K), consistent with a singlet ground state at the low temperature limit, which is common for U^4+^ complexes.^38,39^ Pentavalent **2-U** exhibited an average room temperature χ_M_*T* value of 0.35(4) emu mol^–1^ K (μ_eff_ = 1.7(5) μ_B_). U^5+^ characteristically displays low magnetic moments, with the median room temperature value for χ_M_*T* reported at 0.52 emu mol^–1^ K (μ_eff_ = 2.04 μ_B_).^38^ The ^1^H NMR spectrum of **3-U** supports the closed shell electronic configuration of U^6+^, exhibiting a single sharp doublet (*J*_1H-31P_ = 13.9 Hz, Figure S11) resonance, resembling that of the related closed shell Ce^4+^ complex.^18^

### Electronic Absorption Spectroscopy

**Figure 4:**
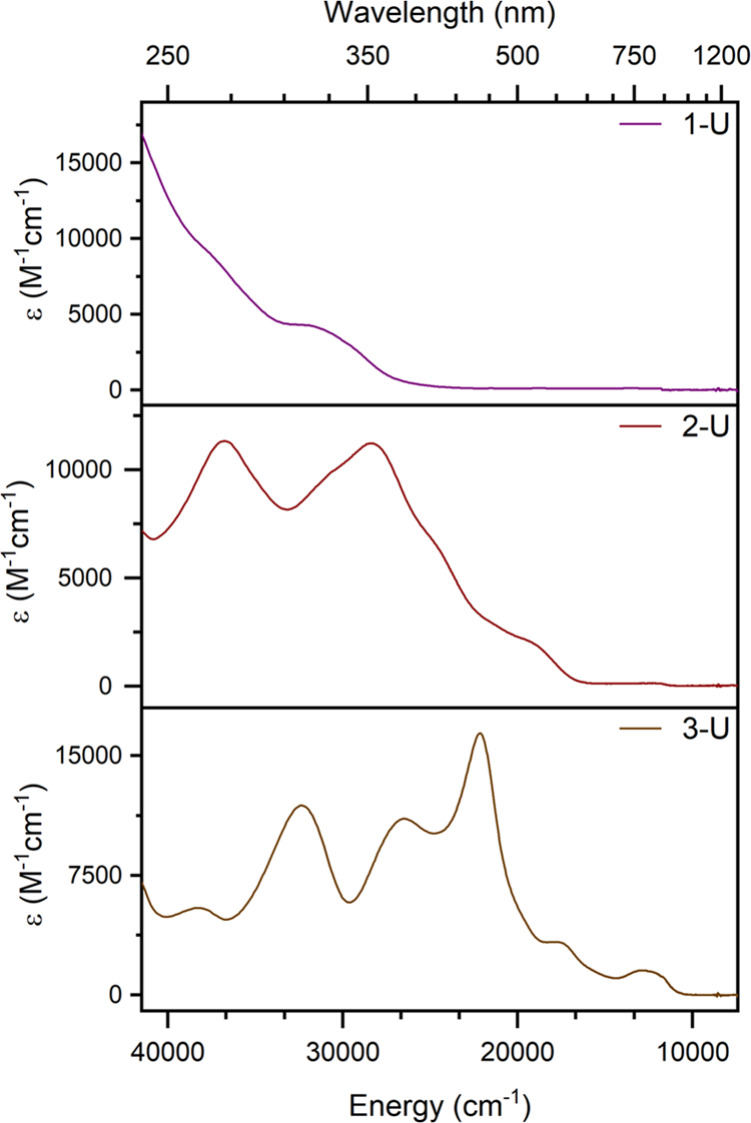
UV–vis-NIR spectra of 1-U (top), 2-U (middle), and 3-U (bottom) in THF at room temperature.

The ultraviolet–visible-near-infrared (UV–vis-NIR) spectra of **1-U**, **2-U**, and **3-U** were collected in THF (Figure 4). The features in all spectra are dominated by intense charge-transfer bands, which occur primarily in the UV region for **1-U**, which is light pink in color, to spanning much of the visible region for **2-U** and **3-U**. **2-U** appears bright red in color, consistent with the local absorption maximum at 352 nm (ε = 11,000 M^–1^cm^–1^), with weaker features extending into the visible region out to approximately 600 nm. The profile of **2-U** is broad; however, multiple features can be visually identified, with local maxima at 272 and 352 nm, and weak 5f-5f transitions are observed between 1200 to 1300 nm (Figure S21), consistent with the related, previously reported^6^ complex, [U^5+^(NP^*t*^Bu(pyrr)_2_)_4_][BArF_20_] (**2-U**^**pyrr**^). The absorption profile of **3-U** immediately after dissolution in THF spans most of the visible region, consistent with its dark brown, inky appearance in solution (Figure 4). The spectrum of **3-U** visually resembles that of **2-U**, with the charge-transfer features shifted to lower energies, suggesting a lower gap between the metal and ligand valence orbitals. Slow degradation of **3-U** occurs in THF at room temperature, which is observable by UV–vis-NIR after 30–40 min in solution. Serial dilution experiments for determination of the ε by linear regression analysis were therefore performed in *ortho*-difluorobenzene (Figure S19).

## Conclusions

Low-coordinate examples of high-valent uranium are uncommon.^3,4^ Bart and co-workers reported the only pseudotetrahedral U^6+^ species, **U**^**NDipp**^, in an alkali metal series of dianionic tetrakis-aryl imido complexes in 2017 (Figure 1).^8^ The geometry of these complexes is noteworthy, as the ligand sterics prohibit the favorable *trans* geometry with respect to other metal–ligand multiple bonds in the actinides, known as the inverse *trans* influence. Depending on the identity of the metal–ligand multiply bonded atom and ligand sterics, the inverse *trans* influence can be too weak to govern the coordination geometry.^8,36,40−43^ While imidophosphorane ligands are monoanionic, their zwitterionic nature distinguishes them from alkoxide and siloxide ligands, resulting in increased stabilization of high-valent ions. This property is exemplified by the divergent metal redox potentials observed in lanthanide imidophosphorane complexes compared to siloxide-supported complexes.^18,20,23,24,26−30^

The significant stabilization of high-valent metal oxidation states by imidophosphorane ligands continues to enable access to lanthanide and actinide complexes in unusually low-coordinate, pseudotetrahedral coordination geometries. This series presents the expansion of uranium complexes in oxidation states largely observed in actinyl or mono-oxo complexes. While less common for uranium, reports of nonactinyl complexes of high-valent neptunium and plutonium are particularly scarce.^19,44−46^ The satisfactory crystalline yields and substantial increases in molecular weight by incorporation of the [BArF_20_]^−^ anion in **2-U** and **3-U** make this system a good candidate for the expansion of high-valent nonactinyl transuranic chemistry, which often requires milligram quantities of metal isotope due to radiological and supply considerations, particularly in the later actinides.

## Experimental Details

### General Considerations

Unless otherwise noted, all syntheses were performed with rigorous exclusion of O_2_/H_2_O using standard Schlenk (UHP Ar) and glovebox (UHP N_2_, <0.1 ppm of O_2_/H_2_O) techniques. Benzyl potassium,^47^ UCl_4_(DME)_2_,^48^ HNP^*t*^Bu_3_,^49^ [Fc][BArF_20_],^50^ and [^*n*^Bu_4_N][BPh_4_]^51^ were prepared as previously described. Further considerations and details can be found in the Supporting Information.

Caution! Depleted uranium is primarily composed of ^238^U, a weak α-emitter (4.197 MeV, *t*_1/2_ = 4.47 × 10^9^ years). Manipulations should be carried out in a ventilated fume hood or glovebox in a lab equipped with appropriate counting equipment.

#### KNP^*t*^Bu_3_ (Modified Procedure)

HNP^*t*^Bu_3_ (898 mg, 4.1 mmol) was dissolved in 10 mL hexanes in a 20 mL scintillation vial, then a glass-coated magnetic stir bar was added. Benzyl potassium (539 mg, 4.1 mmol) was added as a solid in ca. 50 mg portions over 5 min with vigorous stirring. The reaction mixture was stirred overnight to give a white slurry. The white precipitate was collected on a fine-porosity frit and washed with 20 mL *n*-pentane. The solid was transferred to a 20 mL scintillation vial and the residual volatiles were removed *in vacuo* to give the title compound as a white powder (887 mg, 84%). The FTIR spectrum was similar to the previously reported^18^ toluene adduct.

#### [U^4+^(NP^*t*^Bu_3_)_4_] (**1-U**)

UCl_4_(DME)_2_ (216 mg, 1.0 equiv., 0.39 mmol) was massed in a 20 mL scintillation vial subsequently charged with a glass-coated magnetic stir bar and 5 mL Et_2_O. KNP^*t*^Bu_3_ (4.05 equiv., 399 mg, 1.6 mmol) was added as a solid, and any residual material was transferred with 2 mL Et_2_O. The reaction mixture was stirred for 2 d at room temperature, yielding an amber supernatant with a white precipitate. The mixture was filtered through a C porosity frit packed with Celite, and the filter cake was washed with Et_2_O until the filtrate ran clear (20 mL). The volatiles were removed *in vacuo* to give a brown-tinted pink residue. The residue was triturated with 5 × 1 mL *n*-pentane to give the crude product as a crystalline pink solid. The solid was taken up in 10 mL *n*-pentane and filtered through a pipet filter packed with Celite. The light brown solution was concentrated to ∼3 mL *in vacuo* and placed in a–35 °C freezer overnight. The light brown supernatant was decanted from the pink SC-XRD quality crystals (215 mg), then further concentrated to ∼1 mL and placed in a −35 °C freezer overnight. The supernatant was removed to give a second crop of crystals, and the residual volatiles were removed *in vacuo* to give the title compound as a pink crystalline solid (290 mg combined yield, 68%).

^1^H NMR (400 MHz, C_6_D_6_) δ 1.18 (d, *J*_1H-31P_ = 9.6 Hz, 108H).^13^C{^1^H} NMR (101 MHz, C_6_D_6_) δ 50.17 (d, *J*_13C-31P_ = 44.3 Hz), 8.89 (br, fwhm = 27 Hz). ^31^P{^1^H} NMR (162 MHz, C_6_D_6_) δ 369.91 (s). ATR-IR: ν (cm^–1^) = 3003 (w), 2957 (w), 2898 (m), 2866 (m), 1472 (w), 1446 (vw), 1385 (w), 1361 (w), 1187 (m), 1083 (s), 1017 (m), 931 (m), 803 (m), 614 (s), 526 (w), 495 (m), 432 (w). Elemental analysis, C_48_H_108_N_4_P_4_U, % found(calculated): C 52.35(52.25), H 10.16(9.87), N 5.60(5.08).

#### [U^5+^(NP^*t*^Bu_3_)_4_][BArF_20_] (**2-U**)

**1-U** (78 mg, 1.0 equiv., 71 μmol) was massed in a 20 mL scintillation vial subsequently charged with a glass-coated magnetic stir bar and 1 mL THF. [Fc][BArF_20_] (62 mg, 1.01 equiv., 72 μmol) was dissolved in 1 mL THF, then added to the reaction vial with the solution stirred. The reaction mixture immediately changed color from pink to bright red upon addition, and the reaction mixture was stirred for 5 min. The volatiles were then removed *in vacuo* to give a bright red residue, which was washed with 10 mL *n*-pentane to remove ferrocene. The crude product was taken up in 1 mL THF and filtered through a pipet filter, then concentrated to <0.5 mL volume. The concentrated solution was layered with 3 mL Et_2_O, then placed in a −35 °C freezer. After 2 d, the brown supernatant was decanted off and the red crystals were rinsed with 2 × 1 mL Et_2_O. The residual volatiles were removed *in vacuo* to give the title compound as a bright red crystalline solid (103 mg, 82%). Crystals suitable for SC-XRD were grown at −35 °C from Et_2_O (**2-U** is sparingly soluble in Et_2_O).

^1^H NMR (400 MHz, THF-*d*_8_) δ 1.51 (d, *J*_1H-31P_ = 9.3 Hz, 108H). ^11^B{^1^H} NMR (128 MHz, THF-*d*_8_) δ −16.95 (s). ^13^C{^1^H} NMR (126 MHz, THF-*d*_8_) δ 148.38 (BArF_20_), 138.34 (BArF_20_) 75.11 (d, *J*_13C-31P_ = 42.8 Hz), 27.63 (s). ^19^F{^1^H} NMR (471 MHz, THF-*d*_8_) δ −133.03 (s, 8F), −165.44 (t, *J*_19F-19F_ = 19.5 Hz, 4F), −168.85 (t, *J*_19F-19F_ = 19.0 Hz, 8F). ^31^P{^1^H} NMR (162 MHz, THF-*d*_8_) δ 84.65 (s). ATR-IR: ν (cm^–1^) = 3010 (w), 2975 (w), 2911 (w), 2875 (w), 1642 (vw), 1151 (m), 1460 (m), 1391 (w), 1368 (w), 1274 (w), 1182 (w), 1085 (m), 1026 (m), 1004 (s), 974 (s), 936 (m), 808 (m), 771 (m), 755 (m), 727 (vw), 685 (w), 661 (w), 622 (m), 573 (w), 528 (vw), 497 (m), 422 (w). Elemental analysis, C_72_H_108_BF_20_N_4_P_4_U, % found(calculated): C 48.42(48.52), H 6.23(6.11), N 3.14(3.14).

#### [U^6+^(NP^*t*^Bu_3_)_4_][BArF_20_]_2_ (**3-U**)

**1-U** (56 mg, 1.0 equiv, 50 μmol) was dissolved in 1 mL THF in a 20 mL scintillation vial equipped with a PTFE magnetic stir bar. In a separate vial, [Fc][BArF_20_] (87 mg, 2.0 equiv. 0.10 mmol) was dissolved in 2 mL THF, then transferred to the reaction vial with the reaction mixture stirred. The reaction mixture changed color from light pink to brown, and was stirred for 30 min at room temperature. The volatiles were removed *in vacuo*, and the brown residue was washed with 10 mL *n*-pentane to remove ferrocene. The residual *n*-pentane was removed *in vacuo*, then the crude product was taken up in 2 mL *o*-DFB. The brown solution was filtered through a pipet filter into a 4 mL shell vial, then concentrated *in vacuo* to 1 mL volume. The solution was layered with 3 mL 1:1 Et_2_O:*n*-pentane, then placed in a–35 °C freezer. After 5 d, the supernatant was decanted from black needle-like crystals, and the crystals were washed with 1 mL Et_2_O. The residual volatiles were removed *in vacuo* to give the title compound as a black crystalline solid (110 mg, 89%). Crystals suitable for SC-XRD were grown from a THF solution layered with Et_2_O at −35 °C.

^1^H NMR (400 MHz, THF-*d*_8_) δ 1.90 (d, *J*_1H-31P_ = 13.9 Hz, 108H).^11^B{^1^H} NMR (128 MHz, THF-*d*_8_) δ −16.93 (s). ^13^C{^1^H} NMR (101 MHz, THF-*d*_8_) δ 52.38 (d, *J*_13C-31P_ = 39.5 Hz), 30.16 (s). ^19^F NMR (376 MHz, THF-*d*_8_) δ −132.96 (d, *J*_19F-19F_ = 12.7 Hz, 8F), −165.20 (t, *J*_19F-19F_ = 20.3 Hz, 4F), −168.63 (t, *J*_19F-19F_ = 19.2 Hz, 8F). ^31^P{^1^H} NMR (162 MHz, THF-*d*_8_) δ 37.19 (s). ATR-IR: ν (cm^–1^) = 3106 (m), 1643 (w), 1513 (m), 1459 (s) 1395 (m), 1373 (w), 1273 (w), 1178 (w), 1086 (s), 979 (s), 951 (s), 929 (s), 807 (m) 773 (m), 755 (m), 728 (w), 661 (m), 626 (m), 573 (vw), 527 (vw), 496 (m), 431 (vw). Elemental analysis, C_96_H_108_B_2_F_40_N_4_P_4_U, % found(calculated): C 46.45(46.85), H 4.51(4.42), N 2.25(2.28).

## Supporting Information Available

Experimental considerations, NMR and UV–vis-NIR spectra, SQUID, electrochemical, and crystallographic data (PDF)
