# Supplementary material for: U4+/5+/6+ in a Conserved Pseudotetrahedral Imidophosphorane Coordination Sphere
Source: Inorg Chem. 2025 Jan 27;64(5):2489–95. doi: 10.1021/acs.inorgchem.4c04973 (PMC11815820; doi:10.1021/acs.inorgchem.4c04973)
Supplement: Supplementary file 1 — ic4c04973_si_001.pdf [file ic4c04973_si_001.pdf]

Supporting Information for

$U^{4+/5+/6+}$ in a Conserved Pseudo-Tetrahedral Imidophosphorane Coordination Sphere

Andrew C. Boggiano,[†] Julie E. Niklas,[†] Maximilian G. Bernbeck,[†] and Henry S. La Pierre[†,‡,#*]

[†]School of Chemistry and Biochemistry, Georgia Institute of Technology, Atlanta, Georgia 30332-0400, United States

[‡]Nuclear and Radiological Engineering and Medical Physics Program, School of Mechanical Engineering, Georgia Institute of Technology, Atlanta, Georgia 30332-0400, United States

[#]Physical Sciences Division, Pacific Northwest National Laboratory, Richland, Washington 99352, United States

*Email: H.S.L: hsl@gatech.edu

Contents

General Considerations....S2

Nuclear Magnetic Resonance (NMR) Spectroscopy....S4

1-U (^{1}H, $^{13}C\{^{1}H\}$, $^{31}P\{^{1}H\}$, ^{1}H-^{31}P HMBC)....S4

2-U (^{1}H, $^{11}B\{^{1}H\}$, $^{13}C\{^{1}H\}$, $^{19}F\{^{1}H\}$, $^{31}P\{^{1}H\}$, ^{1}H-^{31}P HMBC)....S6

3-U (^{1}H, $^{11}B\{^{1}H\}$, $^{13}C\{^{1}H\}$, $^{19}F\{^{1}H\}$, $^{31}P\{^{1}H\}$, ^{1}H-^{31}P HMBC)....S9

Electronic Absorption Spectroscopy (UV-vis-NIR)....S12

UV-vis-NIR Experimental Details....S16

Fourier-Transform Infrared Spectroscopy....S16

Cyclic Voltammetry....S17

1-U....S17

2-U....S19

3-U....S20

SQUID Magnetometry....S22

Experimental Considerations....S22

Single-Crystal X-ray Diffraction....S25

Experimental and Refinement Details....S26

References....S29

General Considerations

Caution! Depleted uranium is primarily composed of ^{238}U, a weak α-emitter (4.197 MeV, $t_{1/2}$ = $4.47x10^9$ years). Manipulations should be carried out in a ventilated fume hood or glovebox in a lab equipped with appropriate counting equipment.

Unless otherwise noted, all reagents were obtained from commercial suppliers and used as received, and all manipulations were performed with rigorous exclusion of oxygen and water using Schlenk techniques under UHP argon, or in a glovebox (Vigor) under a dinitrogen atmosphere (<0.1 ppm O_2/H_2O). The glovebox is equipped with two -35 ˚C freezers and a cold well. All glassware and cannulas/needles were stored in an oven overnight (>8h) at a temperature of ca. 160˚C prior to use.

Materials: Celite and molecular sieves were heated under vacuum at a temperature >250˚C for a minimum of 24 h. C_6D_6 (Cambridge Isotope Laboratories) was pre-dried over 3 Å molecular sieves then vacuum transferred from purple sodium/benzophenone and stored over 3 Å molecular sieves prior to use. THF-d_8 (Cambridge) was degassed by 3 freeze-pump-thaw cycles and vacuum transferred from purple sodium/benzophenone, then stored over 3 Å molecular sieves prior to use. *n*-Pentane, diethyl ether, toluene, and tetrahydrofuran (THF) were purged with UHP-grade argon (Airgas) and passed through columns containing Q-5/alumina and molecular sieves in a commercial solvent purification system (JC Meyer Solvent Systems, Pure Process Technology). All solvents in the glovebox were stored in media bottles over 10% v/v 3 Å molecular sieves. Methanol was dried by refluxing over magnesium turnings activated with iodine overnight under argon and then distilled and stored over 3 Å molecular sieves. *ortho*-Difluorobenzene was refluxed over CaH_2 overnight under argon, then distilled and stored over 3 Å molecular sieves prior to use. Tri-*tert*-butylphosphine (Strem, >99%) was used as received. Potassium *tert*-butoxide was sublimed prior to use in the synthesis of benzyl potassium. Cargile Type NHV immersion oil was degassed on a Schlenk line by stirring under active vacuum with gentle heating (to facilitate stirring, ca. 40°C) overnight prior to use. Quartz wool (Technical Glass) was leached with 0.1 M oxalic acid/0.5 M H_2SO_4 in DI water in a covered beaker for 3 h at 90 °C, with occasional agitation using a glass stir rod.[1] The quartz wool was collected on a frit and washed with 1 L DI water, then dried under vacuum on a Schlenk line overnight. The quartz wool was further dried in an oven set to 160 °C overnight before use.

Analytical: NMR spectra were obtained on a Bruker Avance III 400 or 500 MHz spectrometer at 298 K, unless otherwise noted. ^1H, $^{13}C\{^1H\}$, and $^{31}P\{^1H\}$ NMR chemical shifts are reported in δ, parts per million. ^1H NMR are referenced to the residual ^1H resonances of the solvent. $^{13}C\{^1H\}$ NMR spectra are referenced to the resonance of the deuterated solvent. $^{31}P\{^1H\}$ spectra are externally referenced to H_3PO_4. Peak position is listed, followed by peak multiplicity, integration value, and proton assignment, where applicable. Multiplicity and shape are indicated by one or more of the following abbreviations: s (singlet); d (doublet); t (triplet); q (quartet); dd (doublet of doublets); td (triplet of doublets); m (multiplet); br (broad). UV-visible-NIR spectroscopy was performed in small-volume screw-cap quartz cuvettes (Starna Scientific) with a 1 cm path length on a Hitachi UH4150 UV-vis-NIR scanning spectrophotometer. ATR infrared measurements were performed on powder samples using a Bruker ALPHA FTIR spectrometer from 400 to 4000 cm^{-1} inside a dinitrogen glovebox. Combustion analysis was performed on samples sealed for transport in glass ampoules using an EAI CE-440 elemental analyzer at the University of Iowa Shared Instrumentation Facility. X-ray structural determinations were performed at the Georgia Institute of Technology X-ray Crystallography Facility on a Bruker D8 Venture diffractometer. Crystals for X-ray analysis were coated in Cargille Type NVH immersion oil inside a glovebox and brought out in a capped 20 mL scintillation vial. Electrochemical data were measured using a Pine WaveDriver 20 Bipotentiostat/Galvanostat. Measurements were performed in a glovebox under an atmosphere of N_2 with a glassy carbon working electrode (3 mm diameter), a bare Ag wire reference electrode in a fritted capillary filled with the corresponding electrolyte solution, and a platinum wire counter electrode at ambient temperature (~25 °C). The fritted capillary was stored in electrolyte solution when not in use, and the inner solution was replaced with

fresh electrolyte solution prior to use. The glassy carbon and Ag wire electrodes were polished before use. Electrolyte solutions were prepared in THF. Measurements were made in positive feedback iR compensation mode (~1200 Ω for 0.05 M [^{n}Bu$_4$N][BPh$_4$] in THF). All potentials are reported vs. Fc^+/Fc, using the conversion[2] of $E^{0\prime}$ of decamethylferrocene = -0.50 V vs. Fc^+/Fc for measurements using [^{n}Bu$_4$N][BPh$_4$], as the Fc^+/Fc couple coincides with the measurement window. Internal referencing was performed by adding a small amount of decamethylferrocene after each run and sweeping the full window at 200 mV/s.

Nuclear Magnetic Resonance (NMR) Spectroscopy

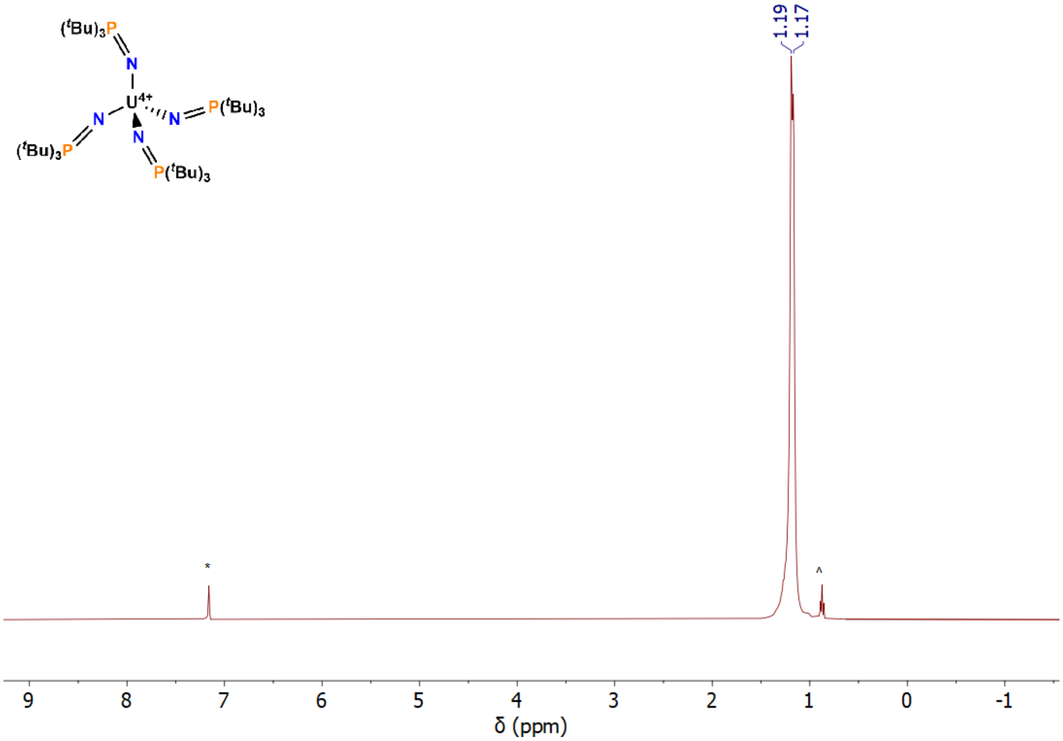

Figure S1. ^{1}H NMR of **1-U** in C_6D_6. * = C_6D_5H, ^ = *n*-pentane, second *n*-pentane resonance overlaps with that of **1-U**.

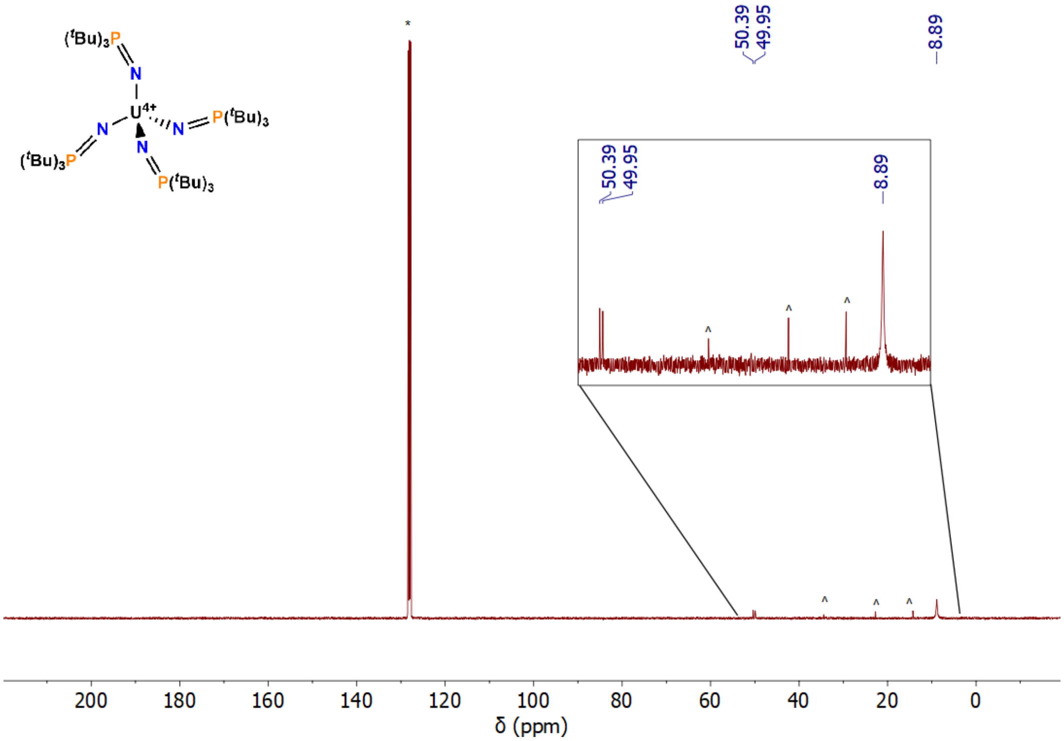

Figure S2. $^{13}C\{^1H\}$ NMR of **1-U** in C_6D_6. * = C_6D_6, ^ = *n*-pentane.

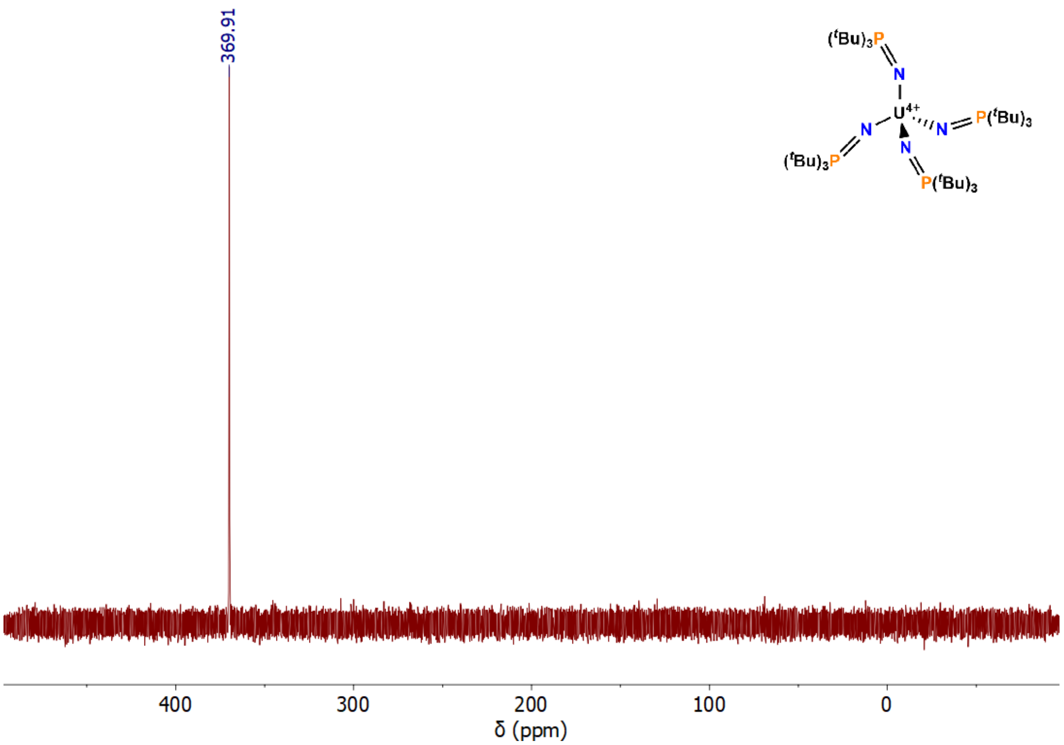

Figure S3. $^{31}P\{^{1}H\}$ NMR of **1-U** in C_6D_6.

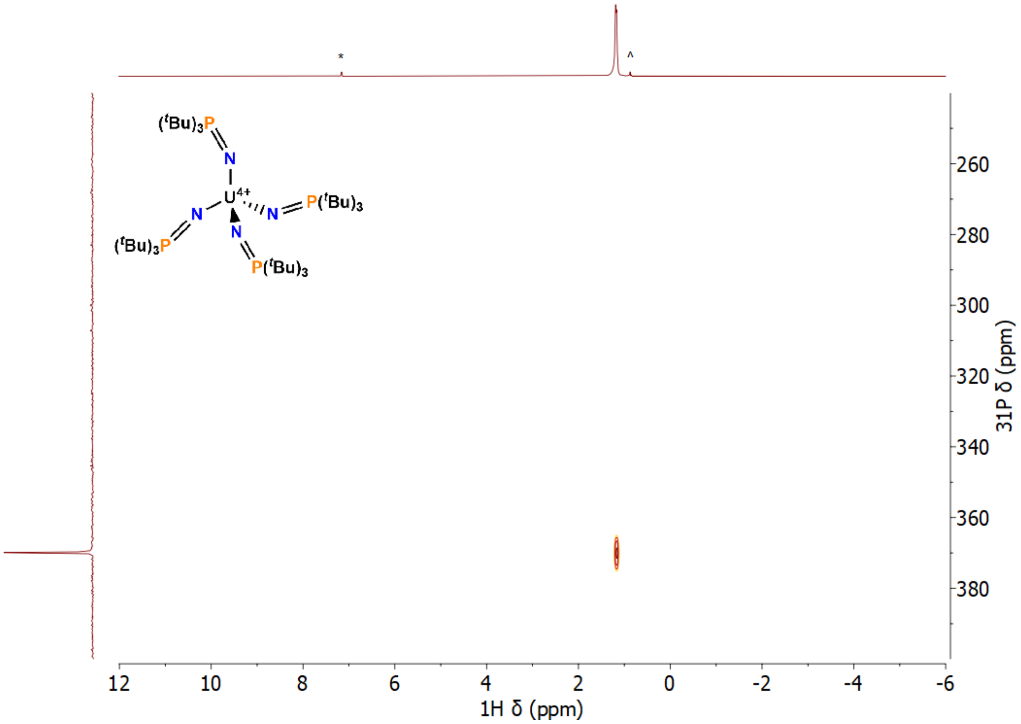

Figure S4. ^{1}H-^{31}P HMBC NMR of **1-U** in C_6D_6. * = C_6D_5H, ^ = *n*-pentane

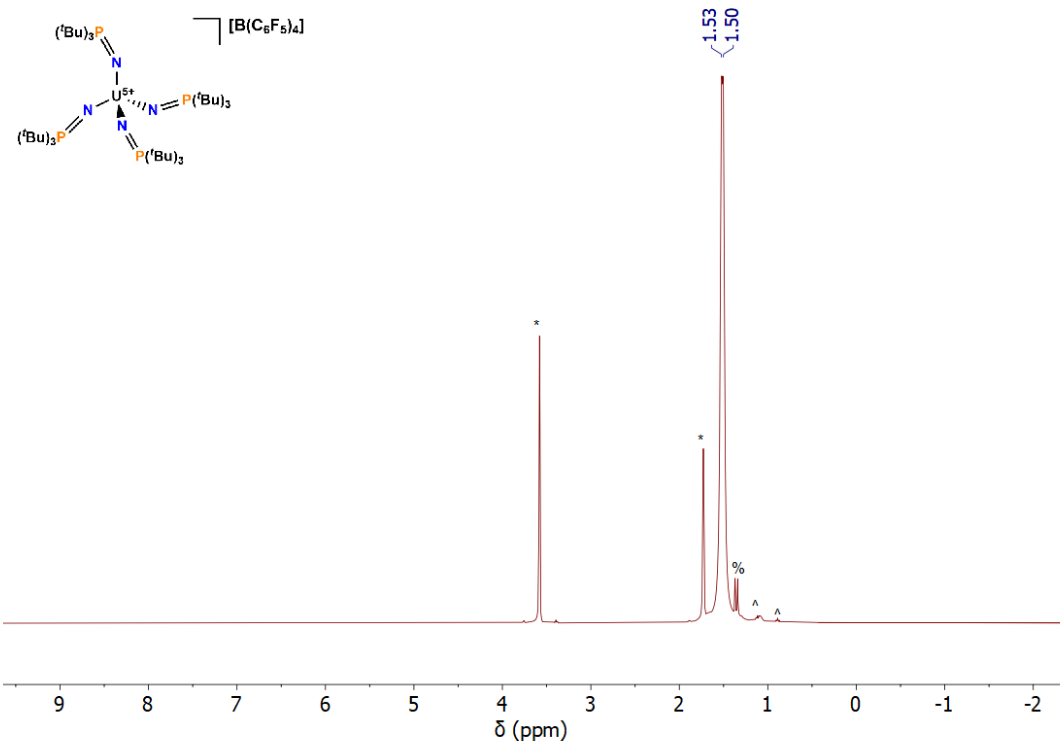

Figure S5. ^{1}H NMR of **2-U** in THF-d_8. * = THF-d_7, ^ = *n*-pentane, % = HNP^{t}Bu$_3$

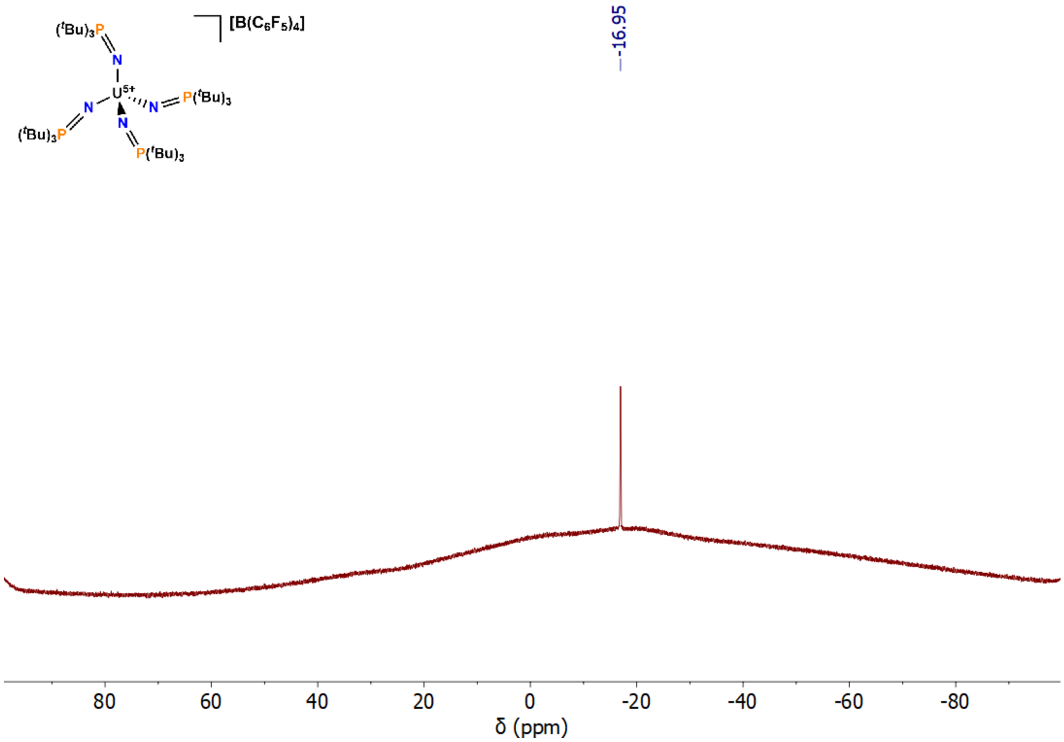

Figure S6. $^{11}B\{^{1}H\}$ NMR of **2-U** in THF-d_8. Background signal is due to NMR probe and borosilicate NMR tube.

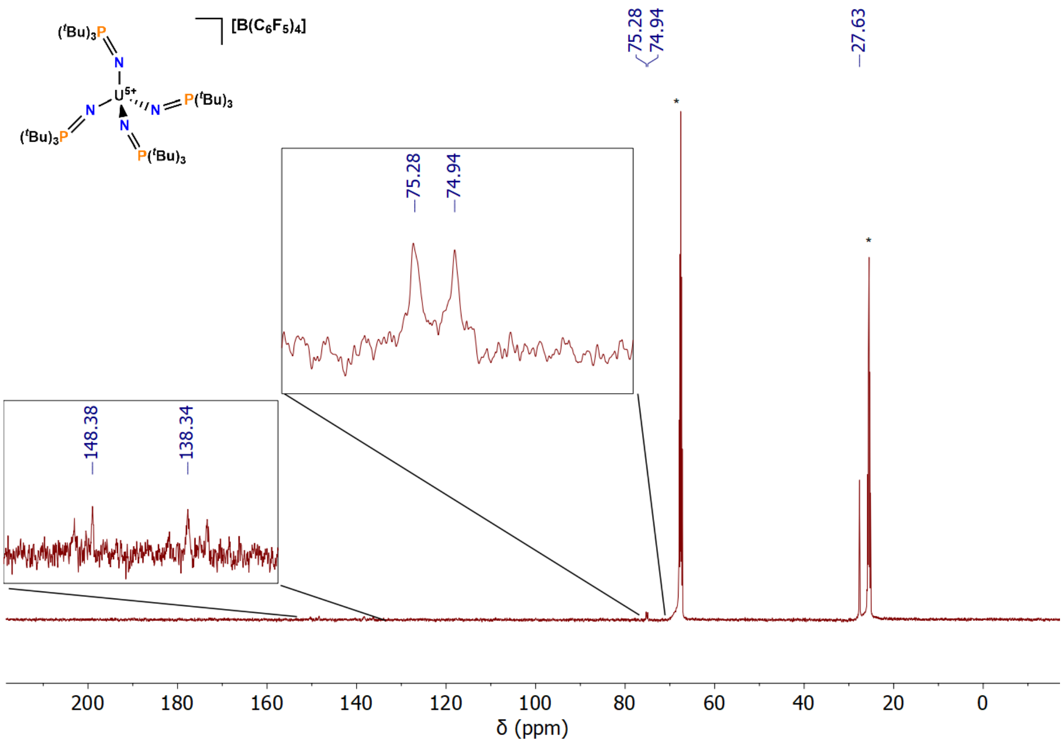

Figure S7. $^{13}C\{^{1}H\}$ NMR of **2-U** in THF-d_8. * = THF-d_8. Note: Not all resonances corresponding to $BArF_{20}$ anion observed as they are quaternary and split by ^{19}F. See ^{11}B and ^{19}F spectra for signals corresponding to $BArF_{20}$.

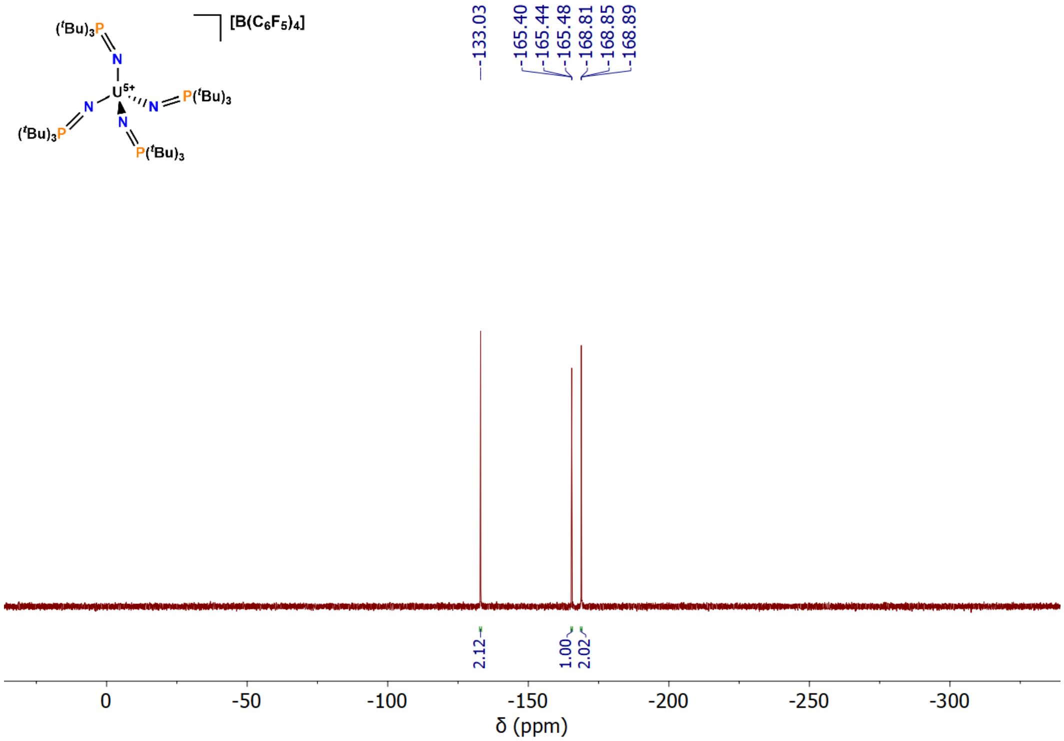

Figure S8. $^{19}F\{^{1}H\}$ NMR of **2-U** in THF-d_8.

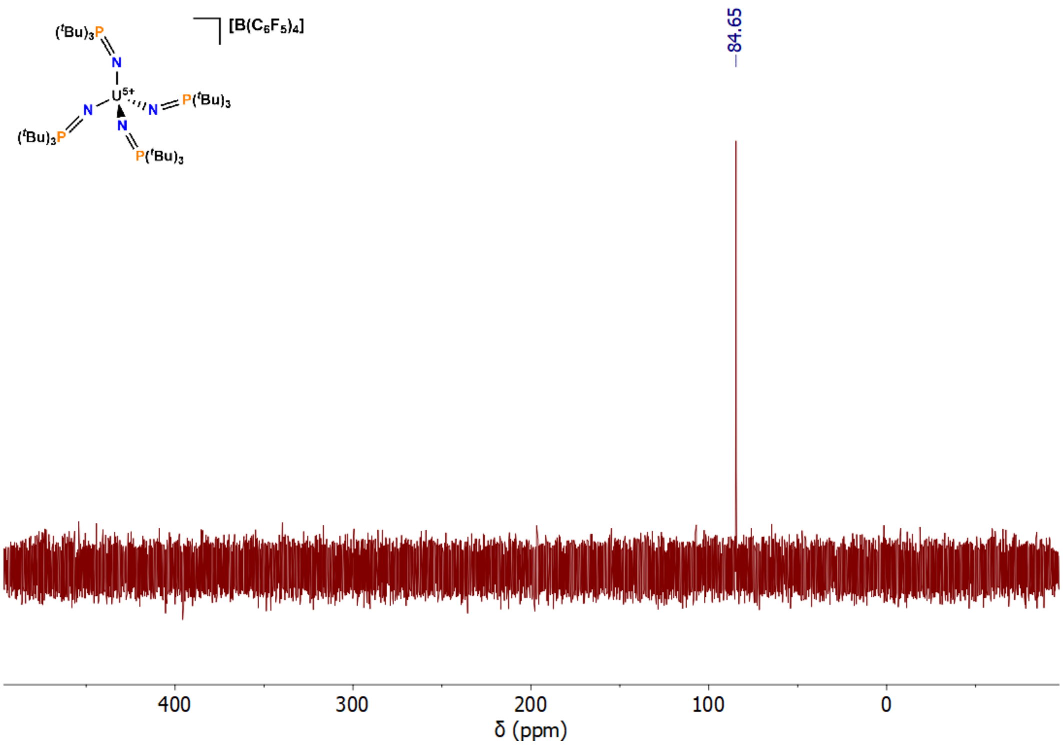

Figure S9. $^{31}P\{^{1}H\}$ NMR of **2-U** in THF-d_8.

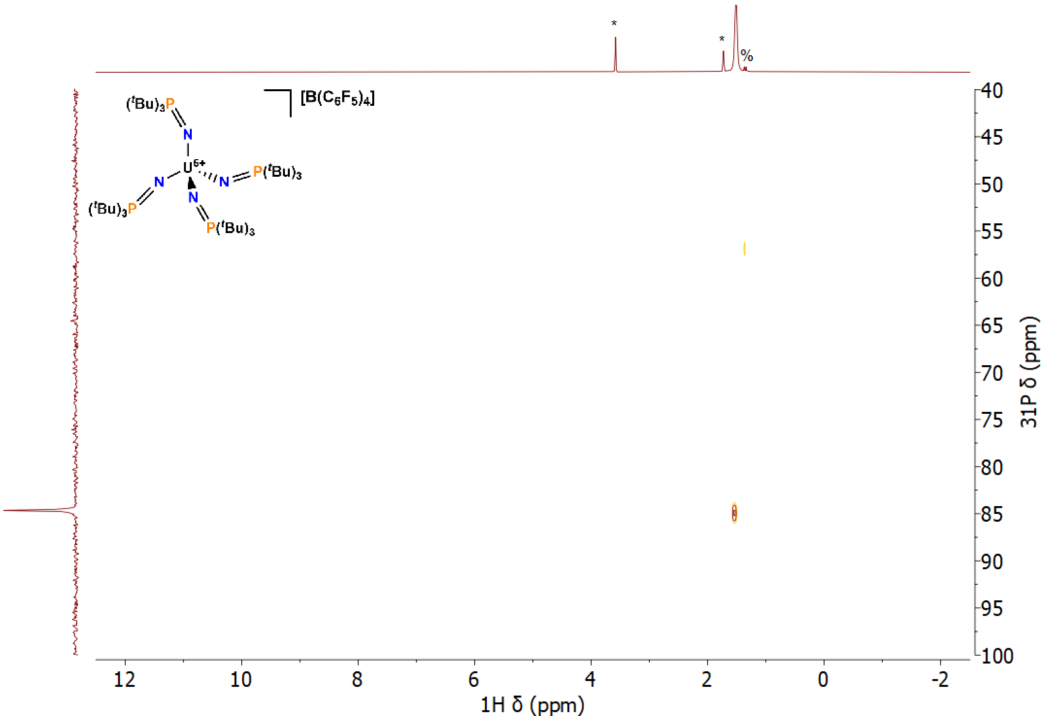

Figure S10. ^{1}H-^{31}P HMBC NMR of **2-U** in THF-d_8. * = THF-d_7, & = HNP^{t}Bu$_3$.

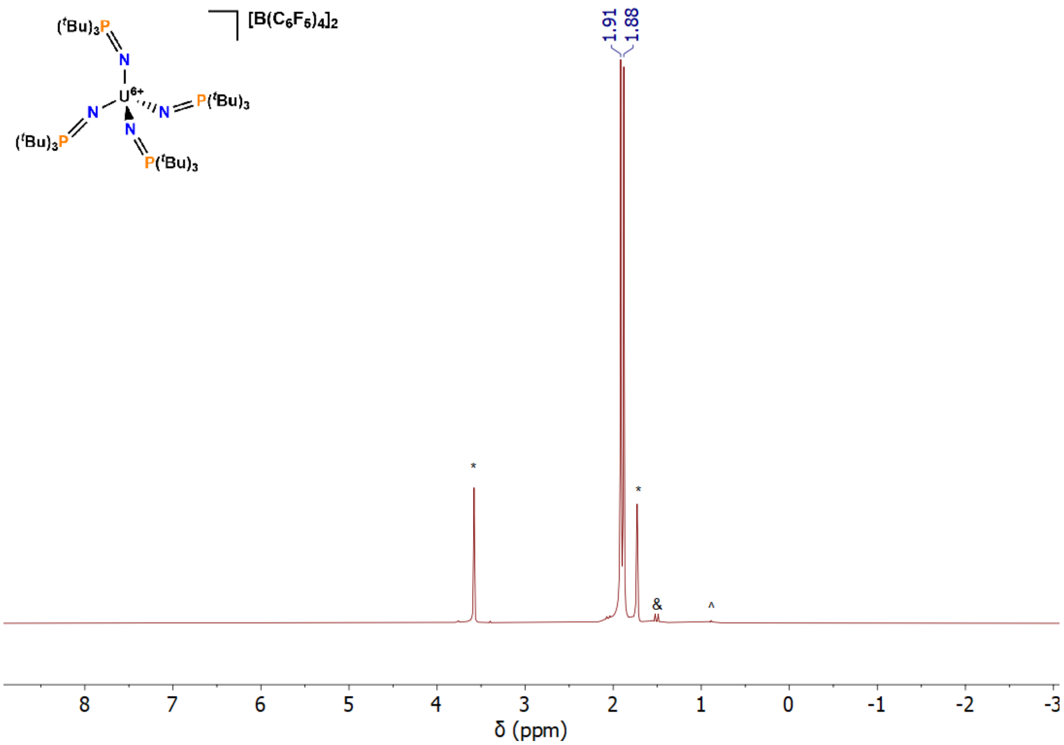

Figure S11. ^{1}H NMR of **3-U** in THF-d_8. * = THF-d_7, ^ = *n*-pentane, & = HNP*t*Bu_3.

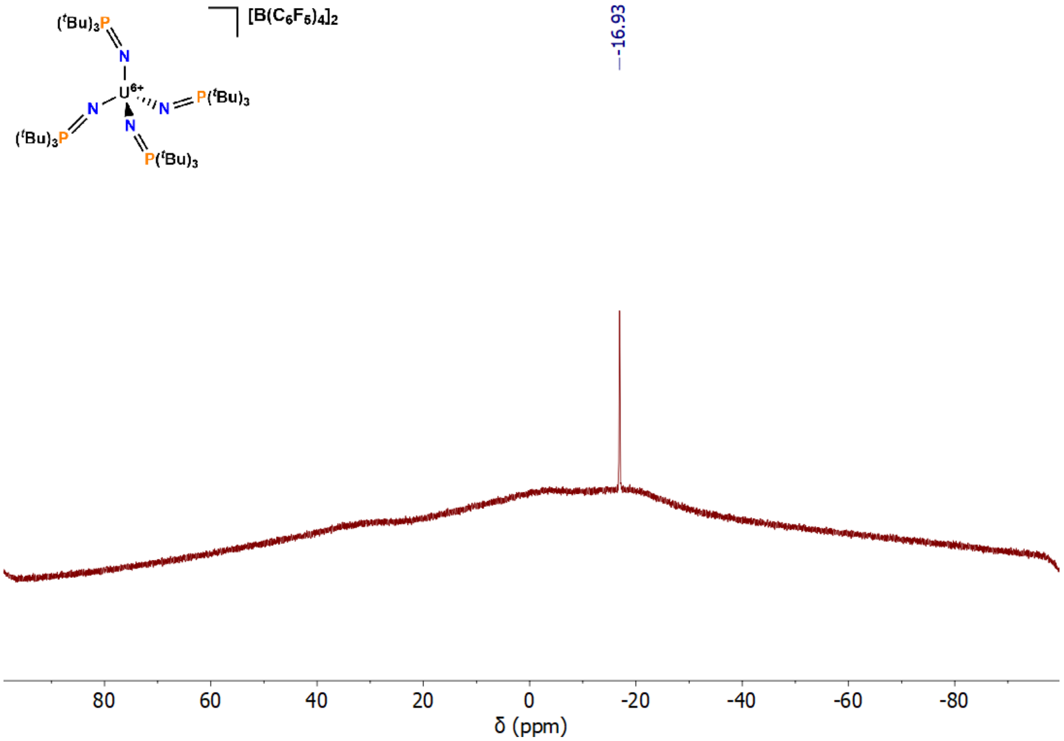

Figure S12. $^{11}B\{^{1}H\}$ NMR of **3-U** in THF-d_8. Background signal is due to NMR probe and borosilicate NMR tube.

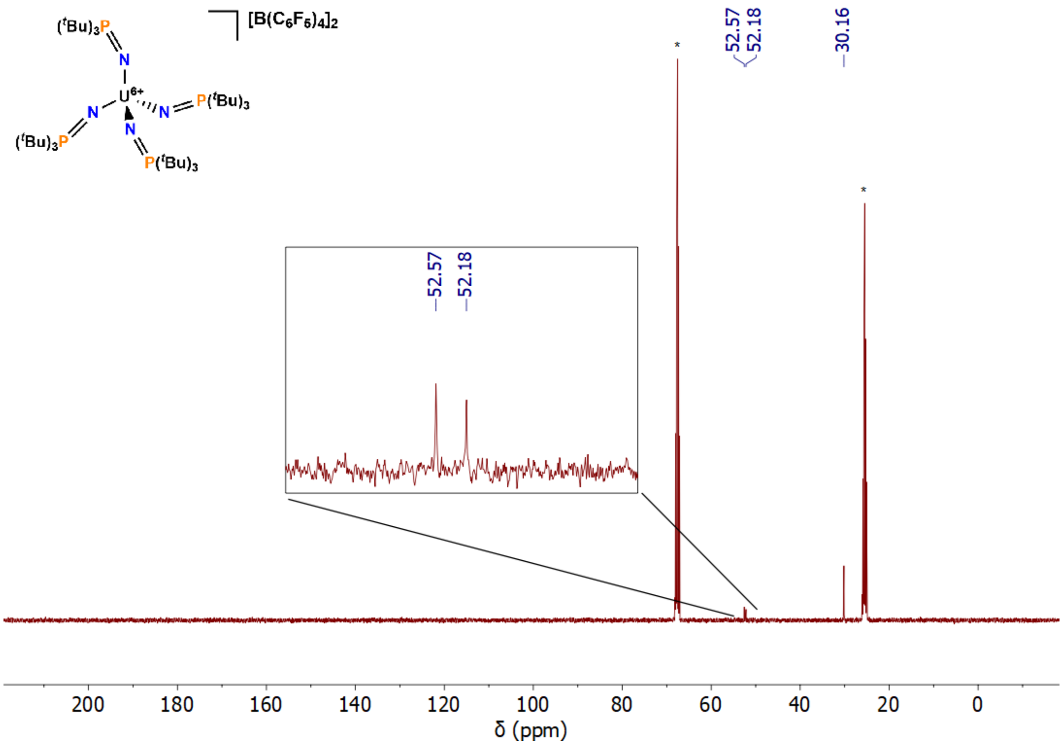

Figure S13. $^{13}C\{^{1}H\}$ NMR of **3-U** in THF-d_8. * = THF-d_8

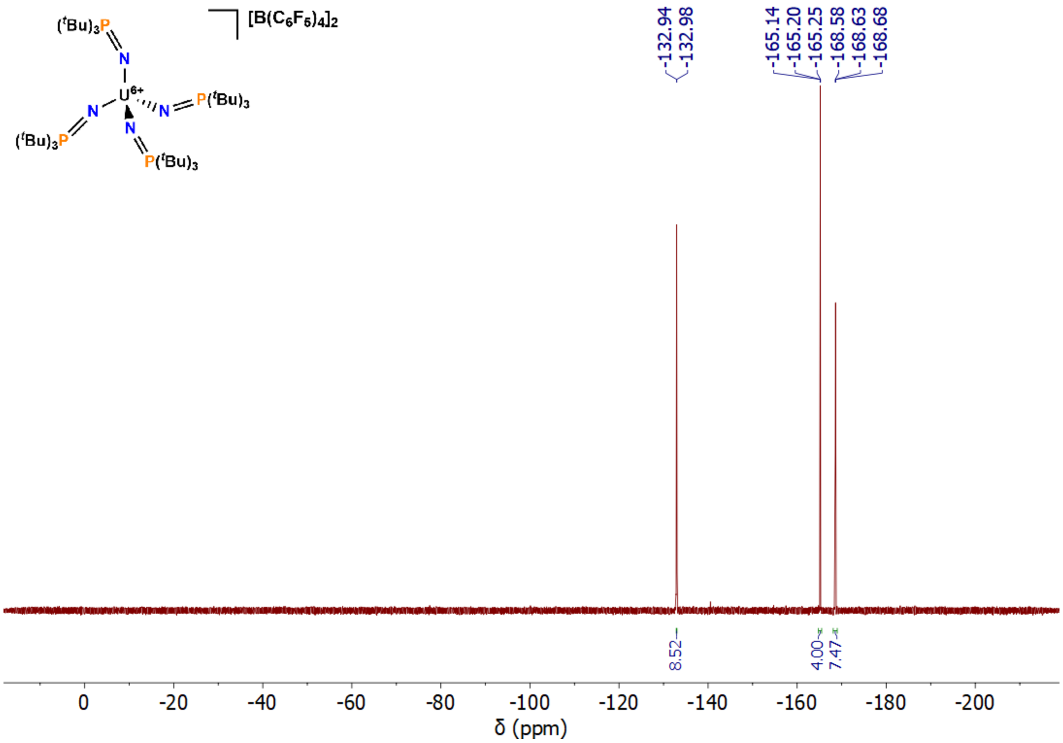

Figure S14. $^{19}F\{^{1}H\}$ NMR of **3-U** in THF-d_8.

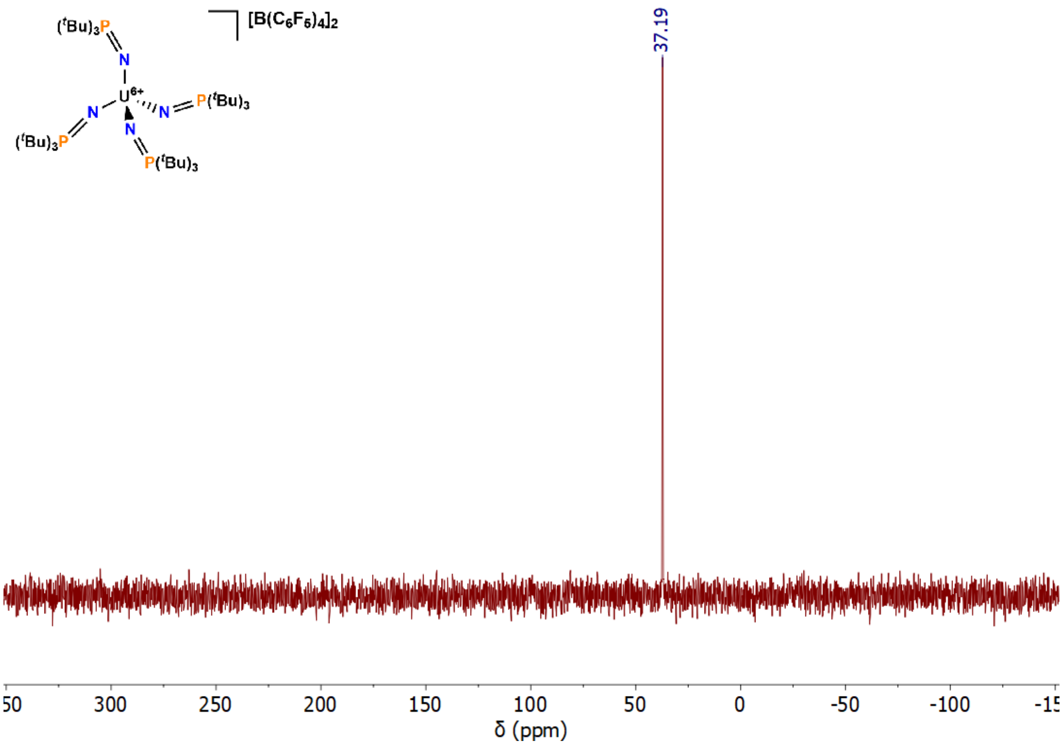

Figure S15. $^{31}P\{^{1}H\}$ NMR of **3-U** in THF-d_8.

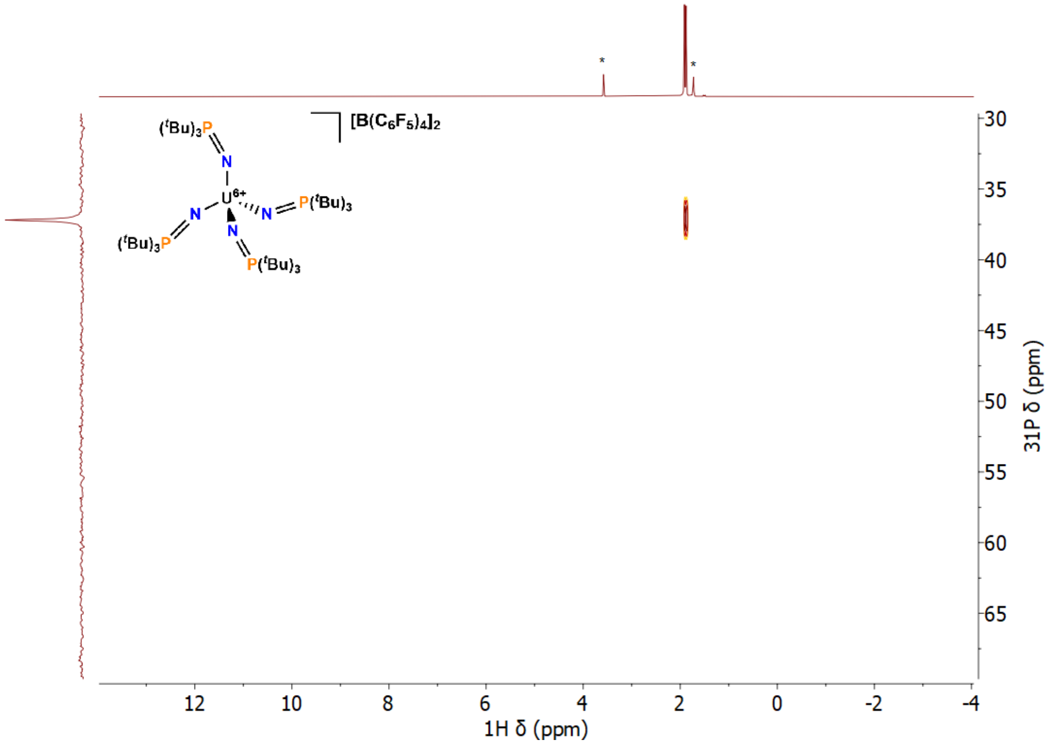

Figure S16. ^{1}H-^{31}P HMBC NMR of **3-U** in THF-d_8. * = THF-d_7

Electronic Absorption Spectroscopy (UV-vis-NIR)

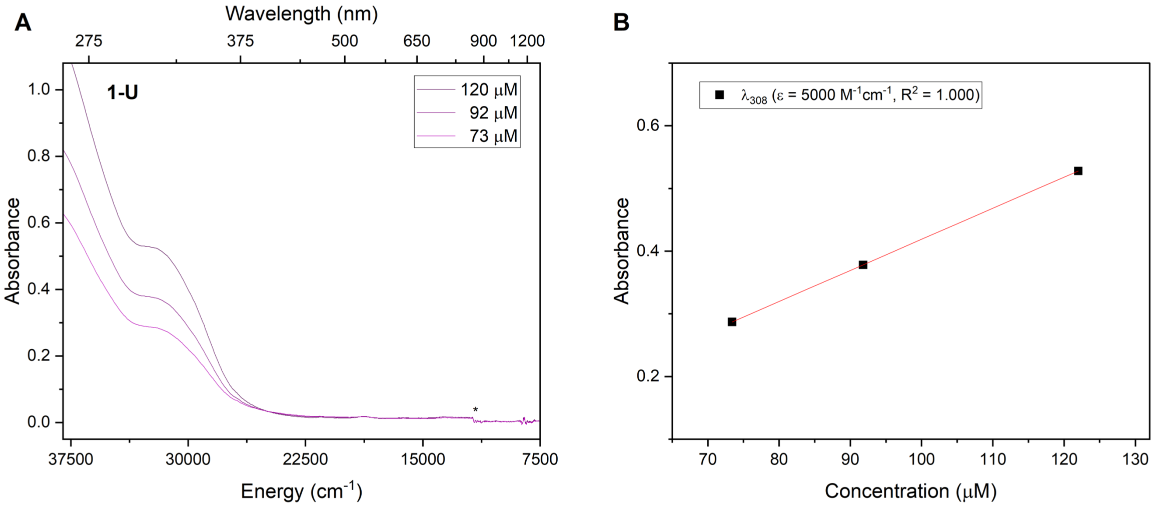

Figure S17. (A) UV-vis-NIR of **1-U** in THF (B) Linear regression analysis to determine ε.

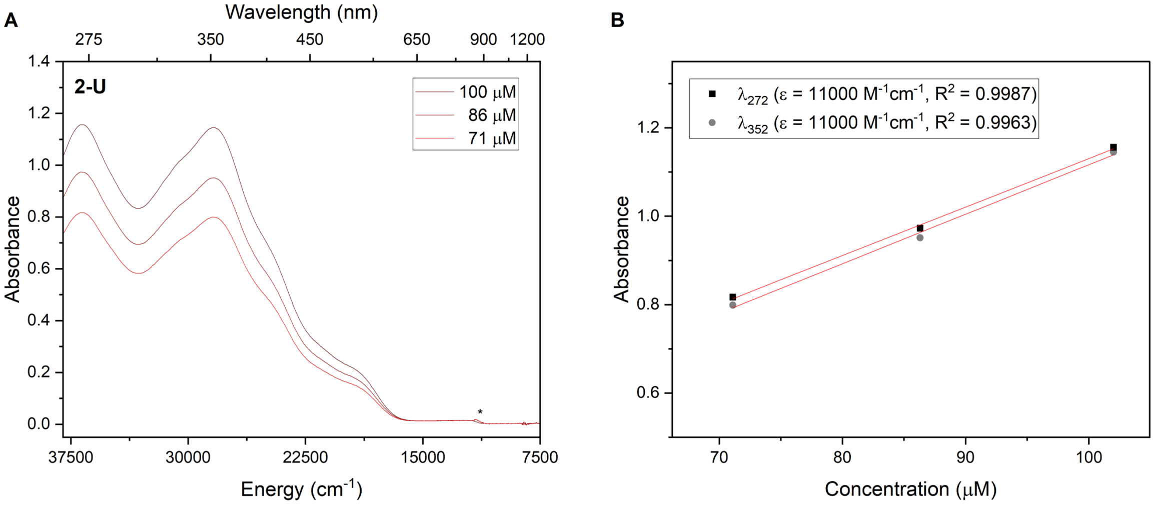

Figure S18. (A) UV-vis-NIR of **2-U** in THF (B) Linear regression analysis to determine ε.

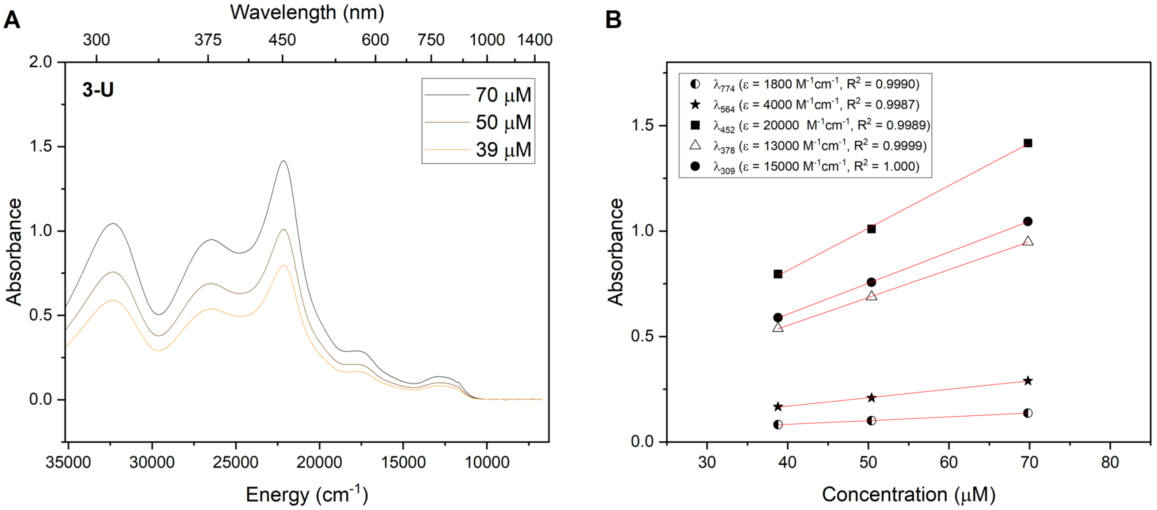

Figure S19. (A) UV-vis-NIR of **3-U** in *o*-DFB (B) Linear regression analysis to determine ε.

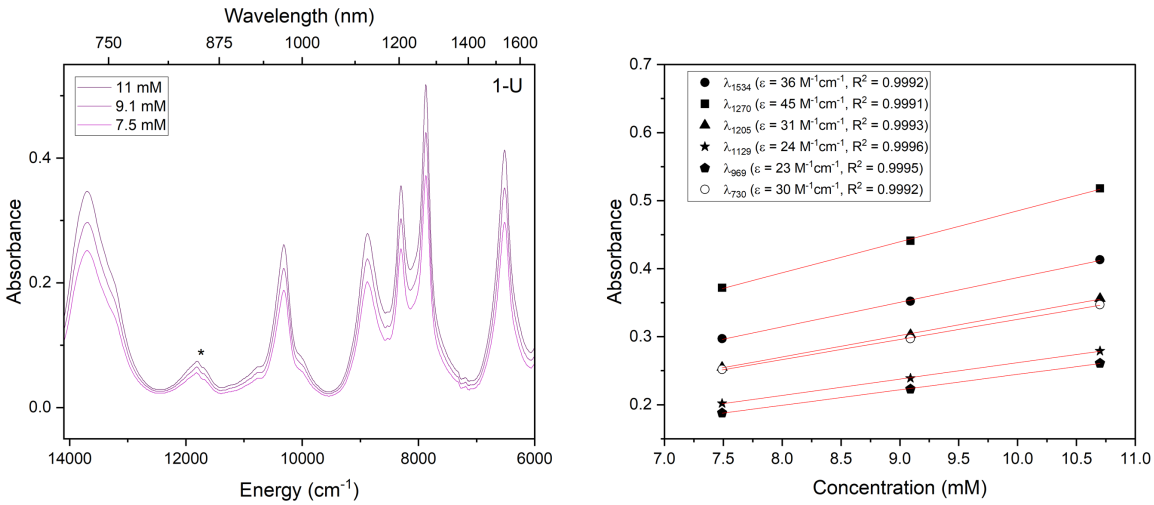

Figure S20. (A) NIR region of **1-U** in THF (B) Linear regression analysis to determine ε of 5f-5f transitions. * denotes detector grating change

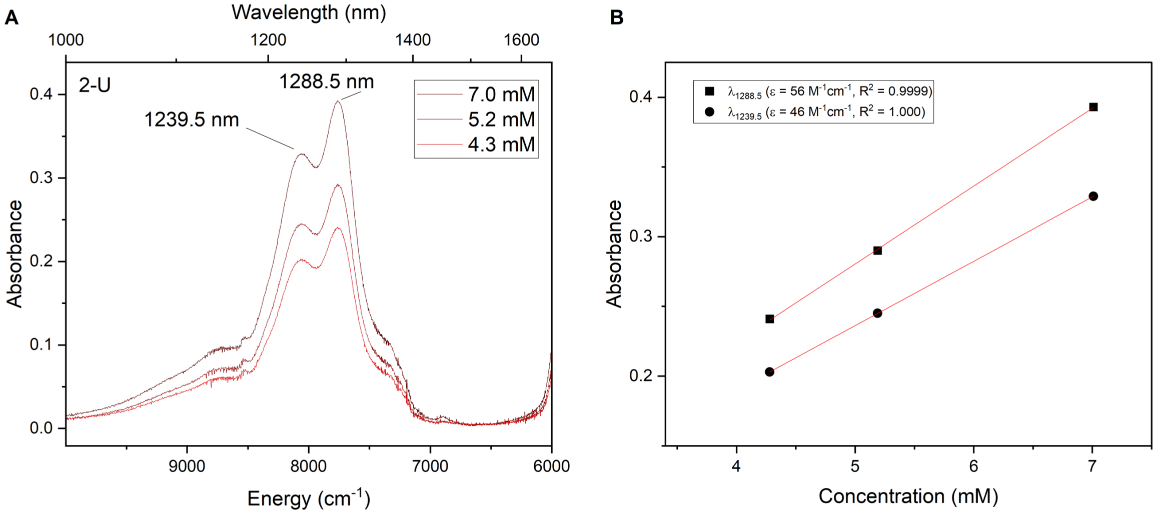

Figure S21. (A) NIR region of **2-U** in THF (B) Linear regression analysis to determine ε of 5f-5f transitions.

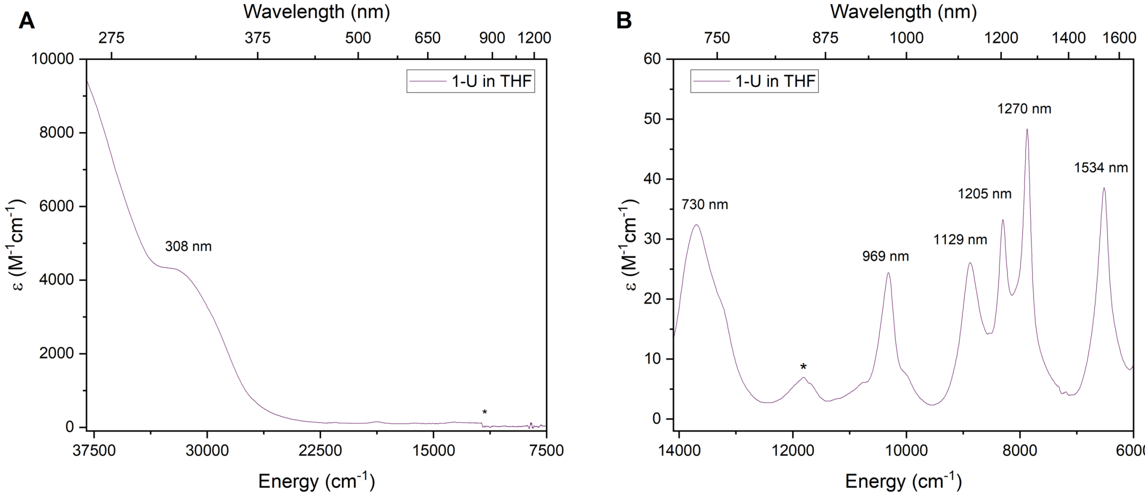

Figure S22. UV-vis-NIR of **1-U** in THF, plotted in ε (A) UV-visible region (B) NIR 5f-5f transitions. * = instrument grating change.

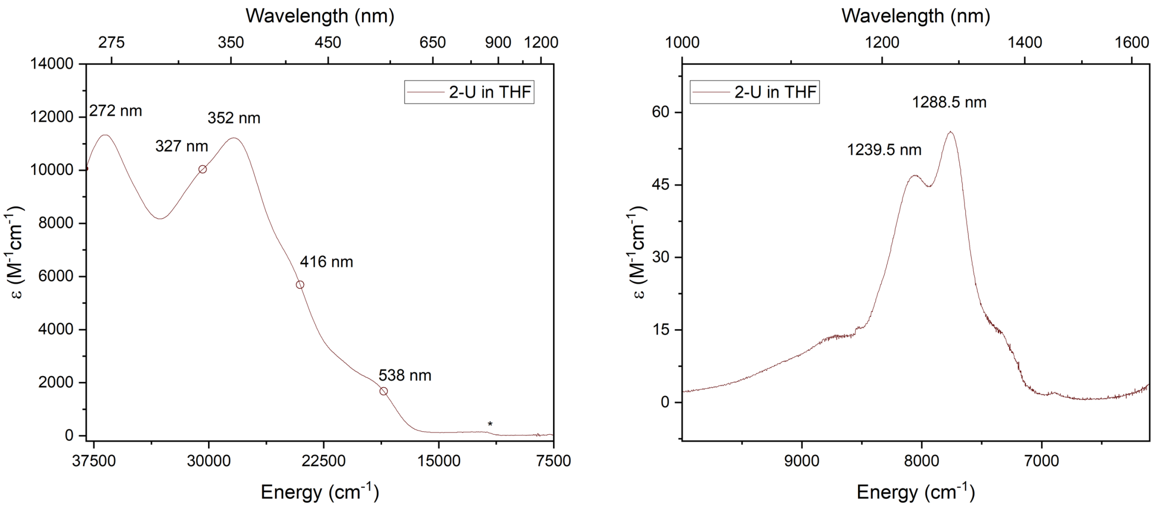

Figure S23. UV-vis-NIR of **2-U** in THF, plotted in ε (A) UV-visible region, inflection points denoted as circles (B) NIR 5f-5f transitions. * = instrument grating change.

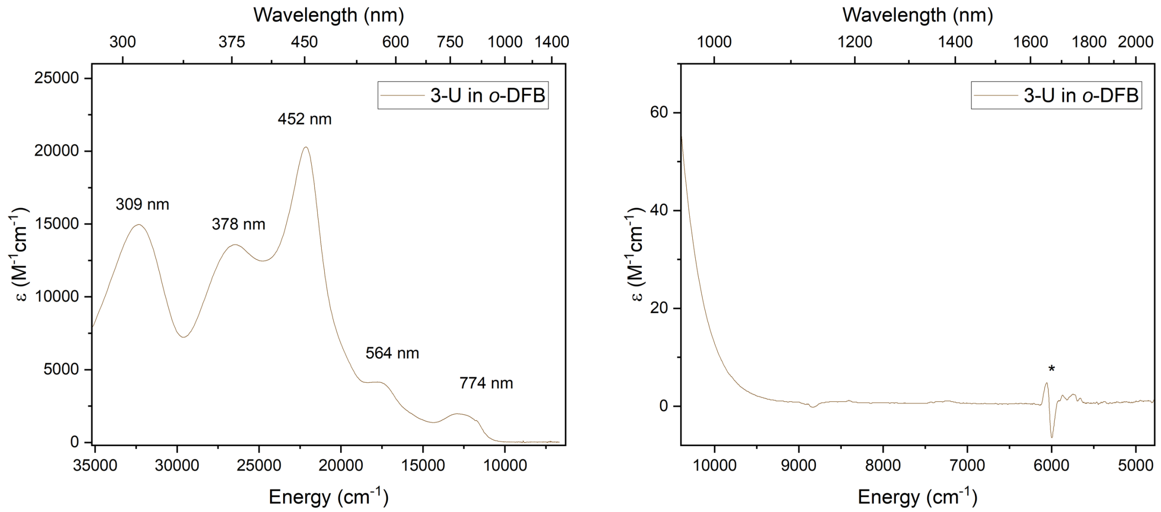

Figure S24. UV-vis-NIR of **3-U** in *o*-DFB, plotted in ε (A) UV-visible region (B) NIR region. * = Artifacts due to first C-H vibrational overtones. Note: solvent window excludes feature at 262 nm observed in THF (below).

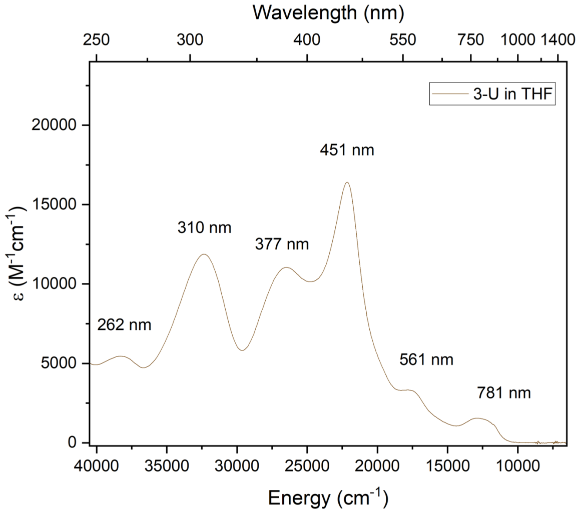

Figure S25. UV-vis-NIR of **3-U** immediately upon dissolution in THF, plotted in ε.

Table S1. Values used for linear regression analysis of UV-vis-NIR absorption features

Compound	Wavelength (nm)	Concentration (μM)	Absorbance
**1-U**	1534	$10.7x10^3$	0.413
		$9.09x10^3$	0.352
		$7.49x10^3$	0.297
	1270	$10.7x10^3$	0.518
		$9.09x10^3$	0.441
		$7.49x10^3$	0.372
	1205	$10.7x10^3$	0.356
		$9.09x10^3$	0.303
		$7.49x10^3$	0.255
	1129	$10.7x10^3$	0.279
		$9.09x10^3$	0.239
		$7.49x10^3$	0.202
	969	$10.7x10^3$	0.261
		$9.09x10^3$	0.223
		$7.49x10^3$	0.188
	730	$10.7x10^3$	0.347
		$9.09x10^3$	0.297
		$7.49x10^3$	0.252
	308	122	0.528
		91.8	0.378
		73.4	0.287
**2-U**	1288.5	$7.01x10^3$	0.393
		$5.19x10^3$	0.290
		$4.28x10^3$	0.241
	1239.5	$7.01x10^3$	0.329
		$5.19x10^3$	0.245
		$4.28x10^3$	0.203
	352	102	1.145
		86.3	0.951
		71.1	0.799
	272	102	1.156
		86.3	0.973
		71.1	0.817
**3-U**	774	69.8	0.137
		50.4	0.101
		38.8	0.082
	564	69.8	0.290
		50.4	0.209
		38.8	0.167
	452	69.8	1.417
		50.4	1.010
		38.8	0.0796
	378	69.8	0.948
		50.4	0.688
		38.8	0.538
	309	69.8	1.045
		50.4	0.757
		38.8	0.589

UV-vis-NIR Experimental Details

A 10 mL stock solution was prepared from isolated crystalline materials using a volumetric flask inside the glovebox. Samples were serially diluted using volumetric flasks and a 1 mL syringe. In the case of 3-U, slow degradation was observed on the time scale of 30-40 minutes. This degradation was significant enough to make acquiring a good linear regression fit difficult for the measurements in THF, and the spectrum presented was collected immediately after preparation of the stock solution. Linear regression analysis was performed in *ortho*-difluorobenzene, in which no degradation was observed. The spectra in THF and *o*-DFB were nearly identical, however, the peak at 262 nm observed in THF is beyond the solvent window for *o*-DFB.

Fourier-Transform Infrared Spectroscopy

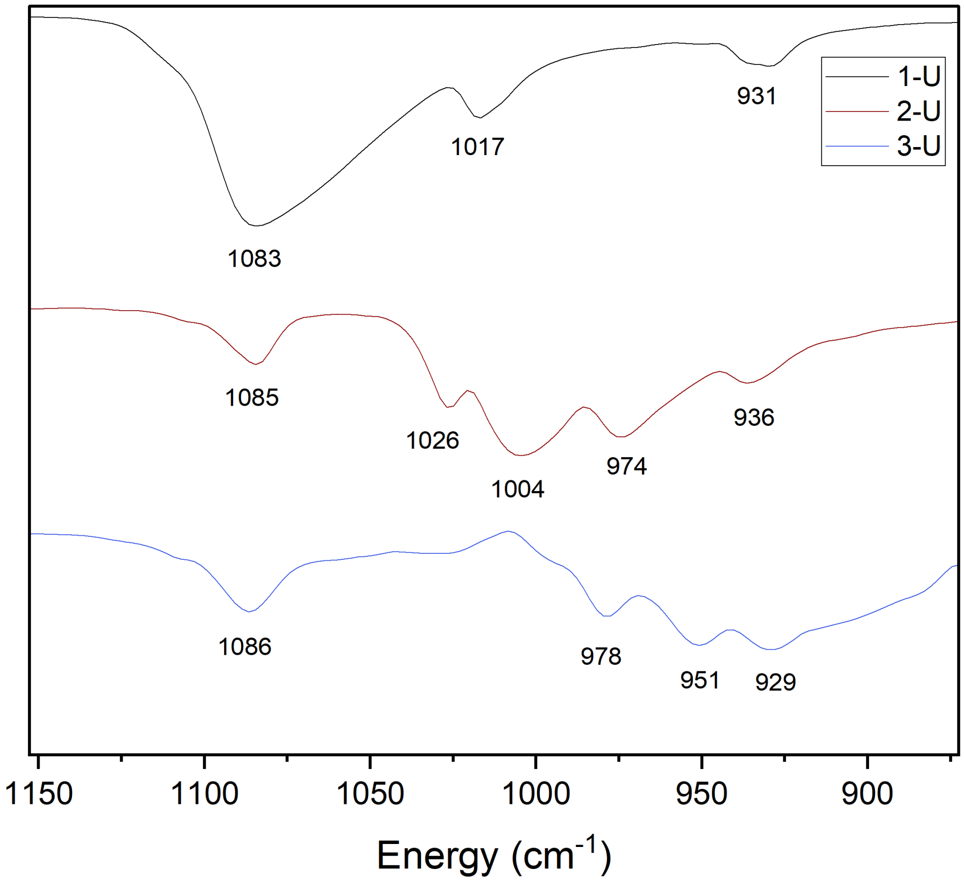

Figure S26. Co-plot of selected region of FTIR spectra of compounds **1-U**, **2-U**, and **3-U**.

Cyclic Voltammetry

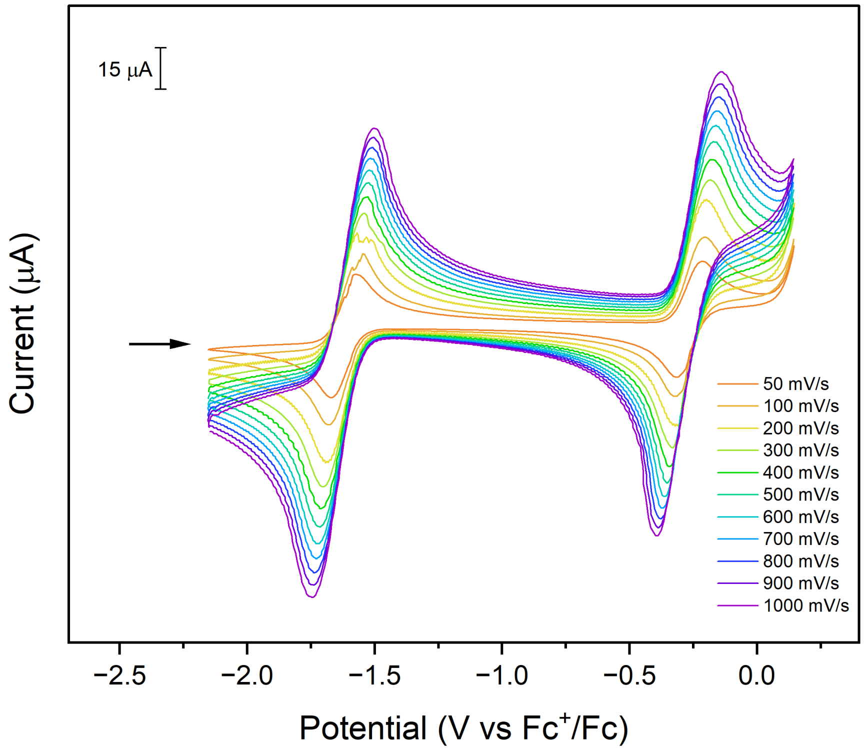

Figure S27. Scan-rate dependence of **1-U** (2.5 mM) in 50 mM [^{n}Bu$_4$N][BPh$_4$] in THF.

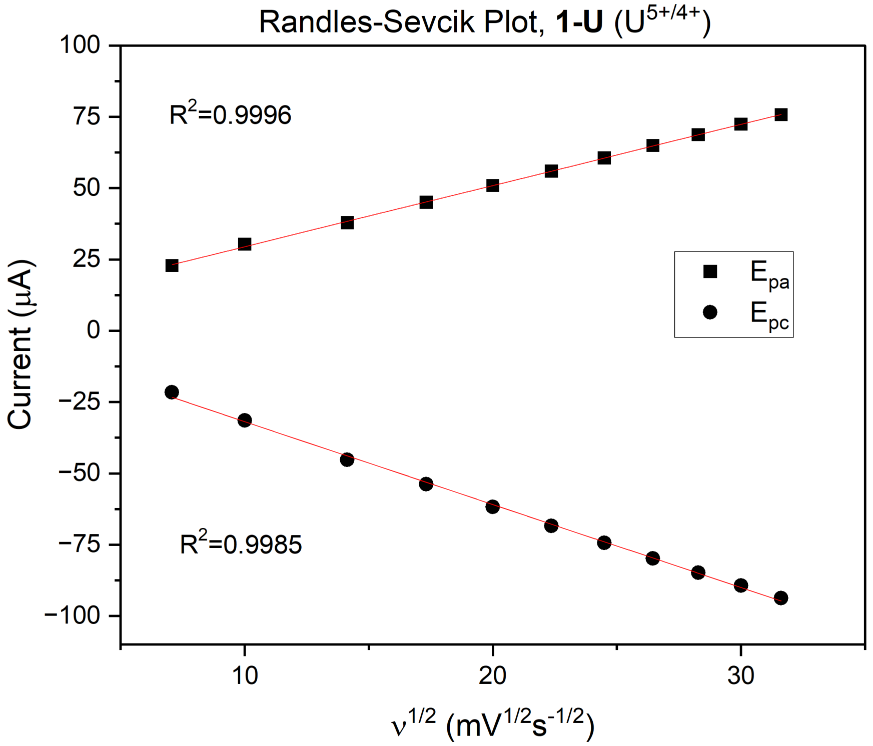

Figure S28. Randles-Sevcik plot of **1-U$^{5+/4+}$** (2.5 mM) in 50 mM [^{n}Bu$_4$N][BPh$_4$] in THF.

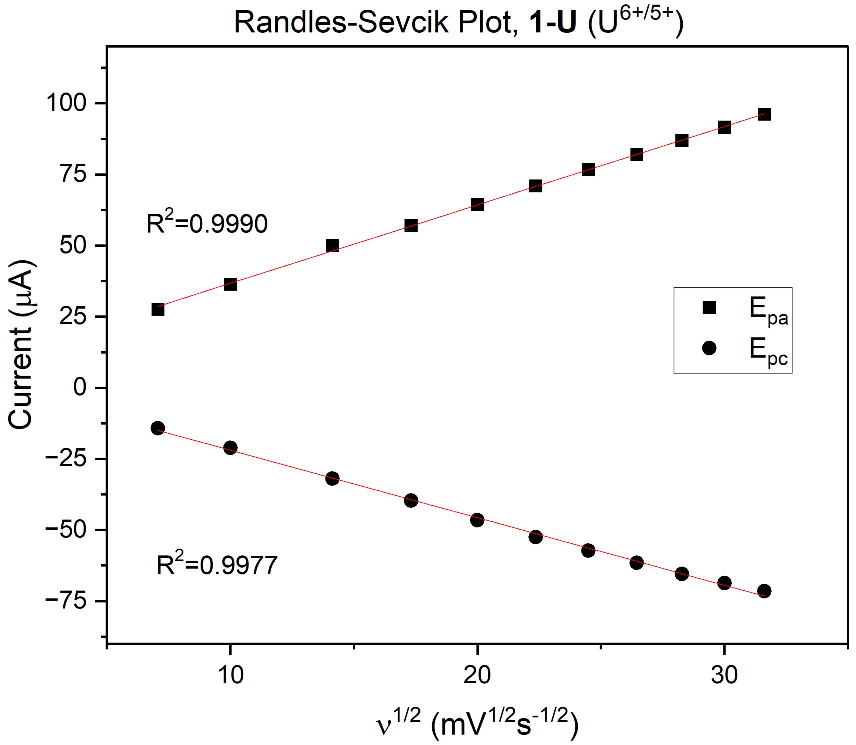

Figure S29. Randles-Sevcik plot of **1-U**$^{6+/5+}$ (2.5 mM) in 50 mM [^{n}Bu$_4$N][BPh$_4$] in THF.

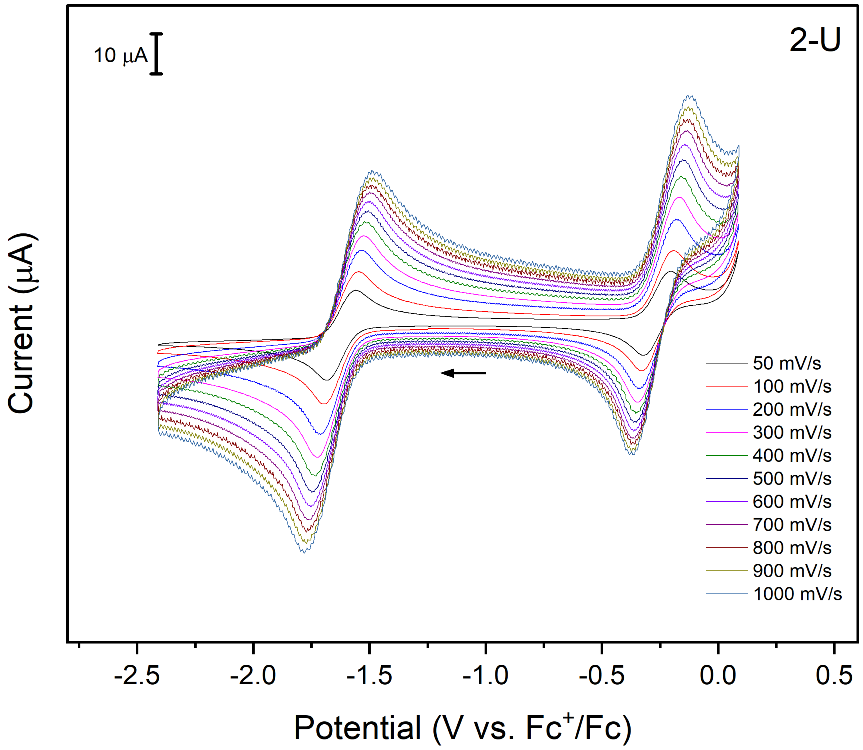

Figure S30. Scan-rate dependence of **2-U** (2.5 mM) in 50 mM [^{n}Bu$_4$N][BPh$_4$] in THF.

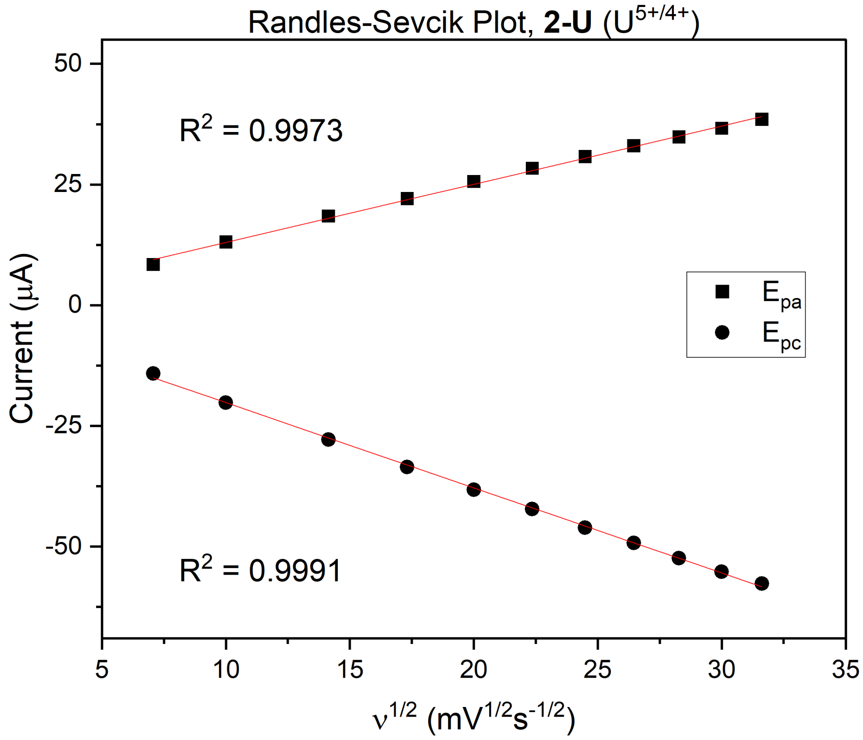

Figure S31. Randles-Sevcik plot of **2-U$^{5+/4+}$** (2.5 mM) in 50 mM [^{n}Bu$_4$N][BPh$_4$] in THF.

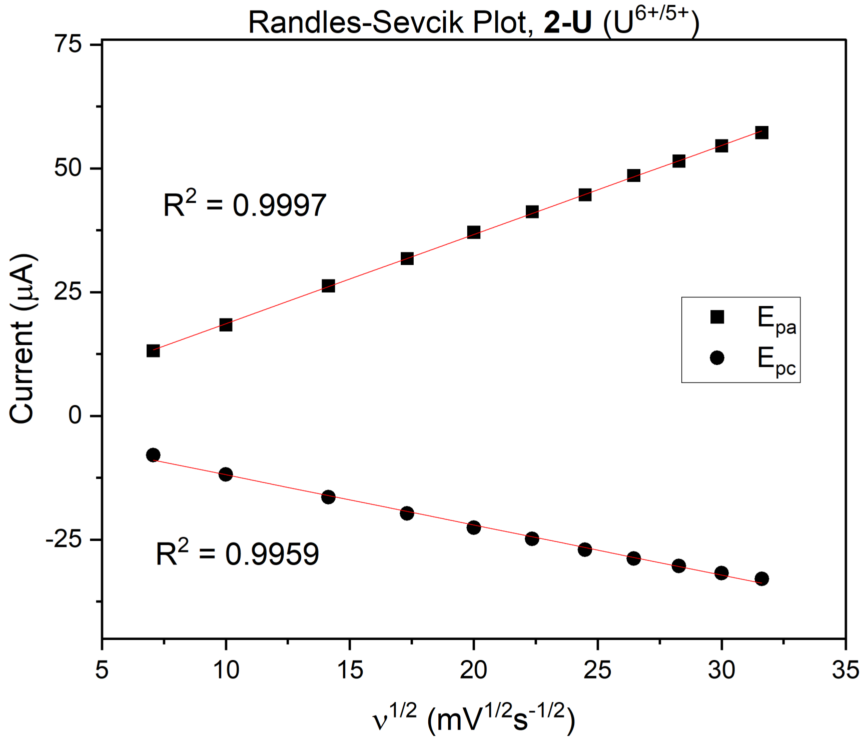

Figure S32. Randles-Sevcik plot of **2-U**$^{6+/5+}$ (2.5 mM) in 50 mM [^{n}Bu$_4$N][BPh$_4$] in THF.

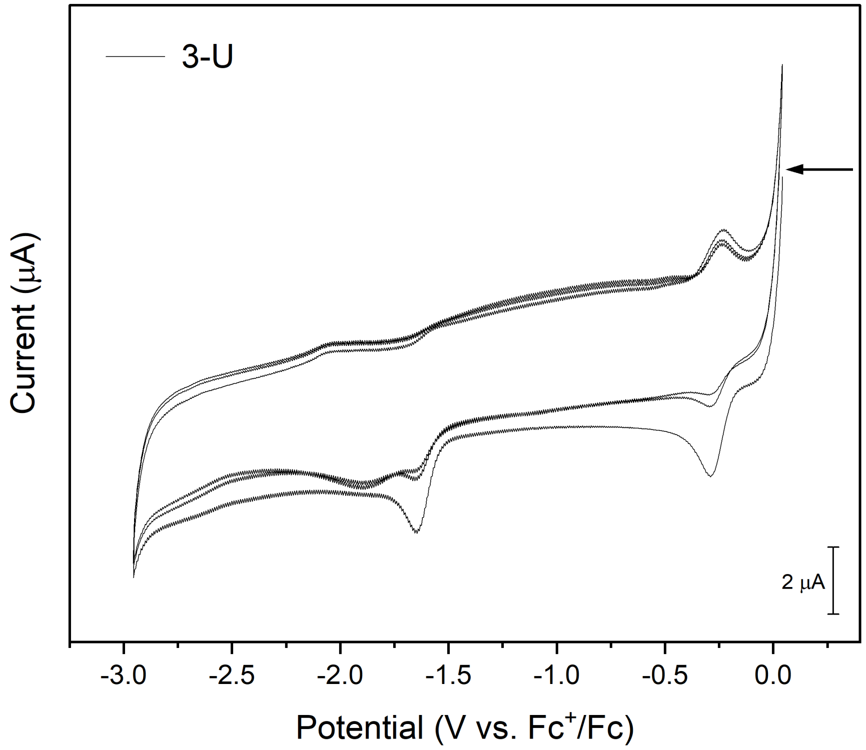

Figure S33. Cyclic voltammogram of heterogeneous mixture obtained after dissolving **3-U** (2.5 mM) in 50 mM [^{n}Bu$_4$N][BPh$_4$] in THF at 200 mV/s scan rate.

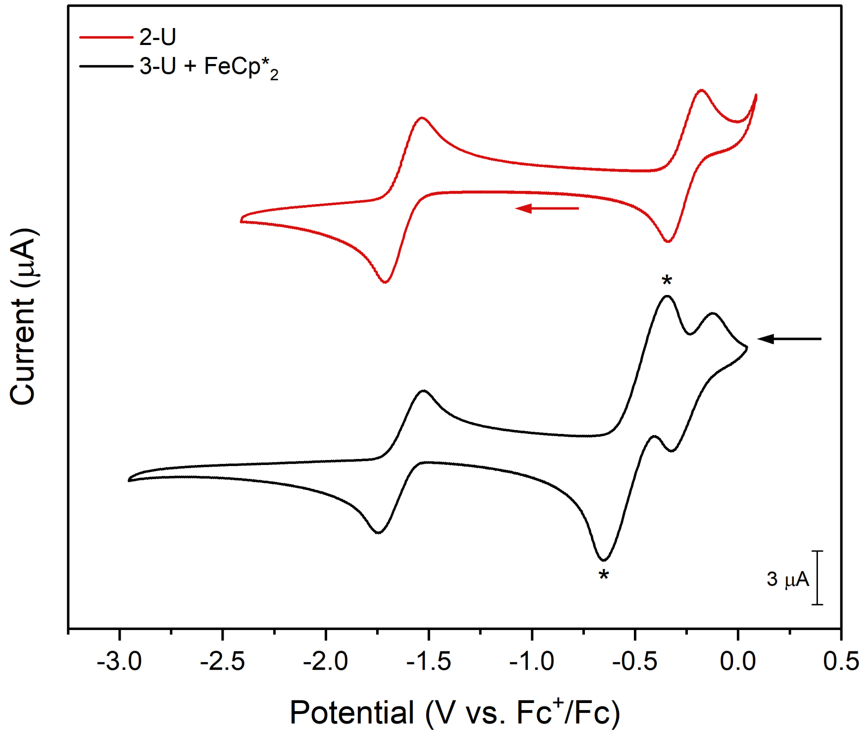

Figure S34. Comparison between cyclic voltammograms of **3-U** after adding $FeCp^*_2$ reference (black), and isolated **2-U** (red). Scan rate = 200 mV/s in 50 mM [^{n}Bu_4N][BPh_4] in THF with analyte concentration 2.5 mM.

SQUID Magnetometry

Experimental Considerations

Magnetic measurements were performed on a Quantum Design MPMS3 magnetometer. Samples were prepared inside of a glovebox. A measured amount of quartz wool was packed on top of the sample with a PTFE rod to immobilize the sample due to its solubility in eicosane. While **2-U** does not exhibit solubility in eicosane, quartz wool was used for consistency with **1-U** and due to the much lower diamagnetic contribution of quartz wool compared to eicosane, which we observed to facilitate more reproducible data for **2-U**. The tops of the sample tubes were fitted with an Ultra Torr Swagelok adaptor, sealed, and transported to a Schlenk line where the tube was evacuated and sealed using a O_2/H_2 torch. The sealed tubes were wrapped with PTFE tape near the top to provide a compression fit and then pressed into plastic straws, which were then loaded into the instrument. Diamagnetic corrections for the quartz wool and complexes were performed using Pascal's constants and are tabulated in table S2.[3] Variable temperature susceptibility measurements were performed from low to high field to reduce remnant magnetic fields in the instrument. The sample was warmed to 300 K between each measurement to thermalize and prevent possible hysteretic effects. Zero field cooled (ZFC) measurements were taken from low to high temperature after cooling with no applied field. Variable field magnetization measurements were recorded after the susceptibility measurements to reduce remnant magnetic fields. The sample was thermalized to 300 K between each measurement and cooled with no applied field. The field was then swept from 0 to +7 T to prevent possible hysteretic effects.

Table S2. Diamagnetic corrections

Compound	Diamagnetic Correction
**1-U**	$-766.92*10^{-6}$ emu/mol
**2-U**	$-1046.28*10^{-6}$ emu/mol
Quartz wool	$-4.16*10^{-10}$ emu/mg
$[NP(^{t}Bu)_3]^-$	$-182.98*10^{-6}$ emu/mol
$[BarF_{20}]^-$	$-288.36*10^{-6}$ emu/mol

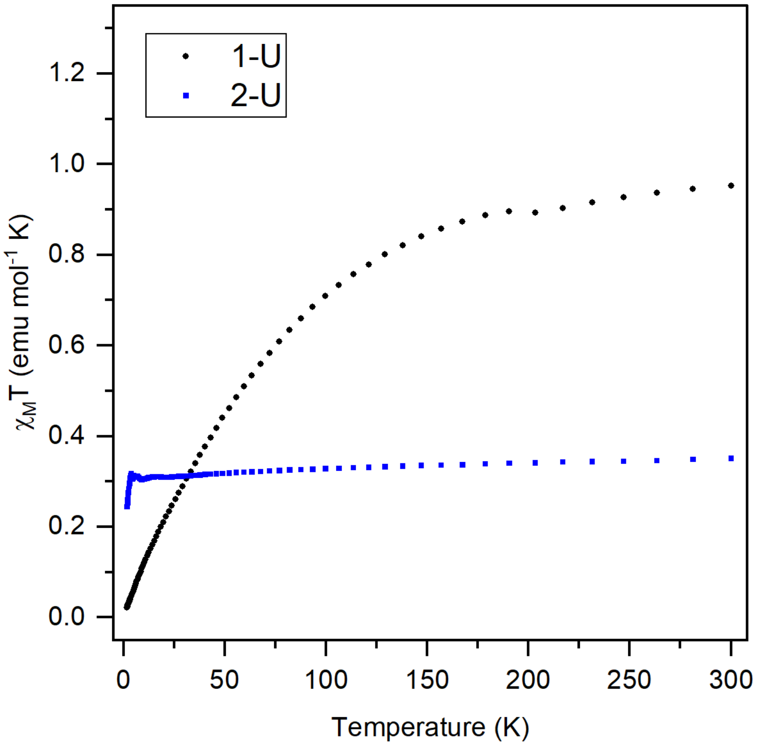

Figure S35. Co-plot of $\chi_M T$ data for **1-U** and **2-U** under 1T dc applied field.

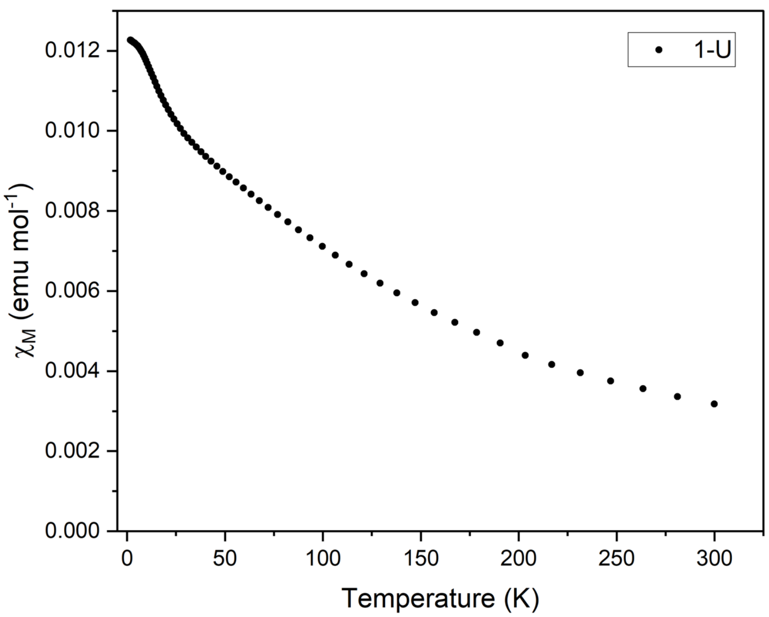

Figure S36. Molar susceptibility data vs. temperature for **1-U** under a dc applied field of 1 T.

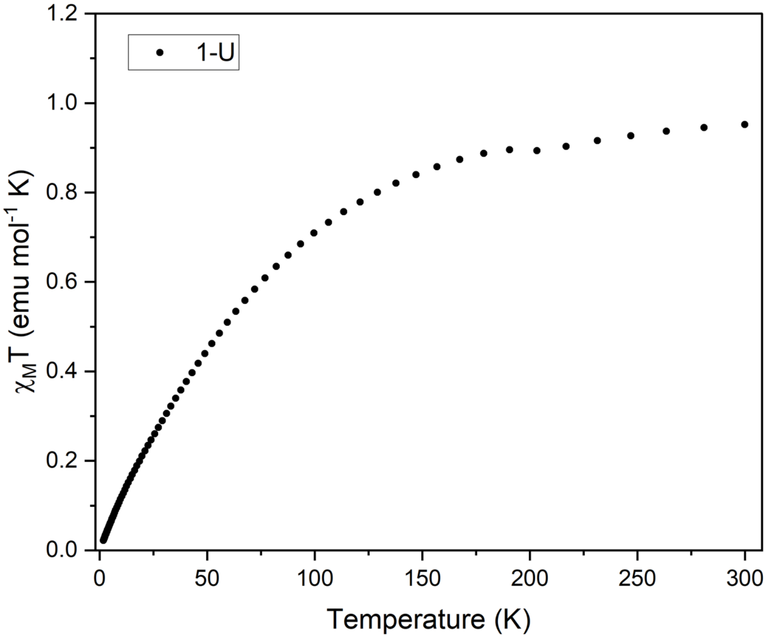

Figure S37. χ_MT vs. temperature for **1-U** under a dc applied field of 1 T.

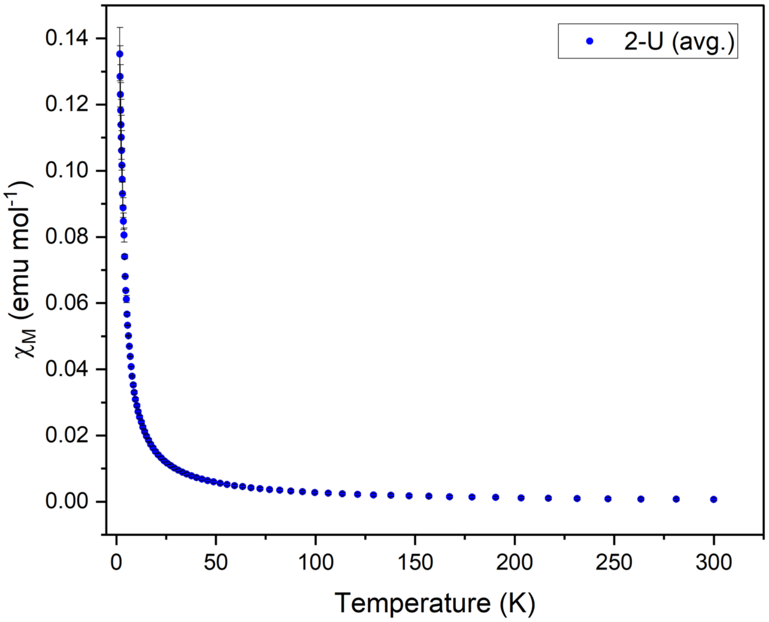

Figure S38. Molar susceptibility data vs. temperature for **2-U** under a dc applied field of 1 T. Note: Upon measurement of individually prepared samples of **2-U** from separate batches, some variation was observed. This is attributed to the overall low moment of the sample, in addition to its large molar mass and diamagnetic contribution. The shape and trends observed were consistent across samples, and the data presented are an average of two independent samples, with error bars representing the uncertainty.

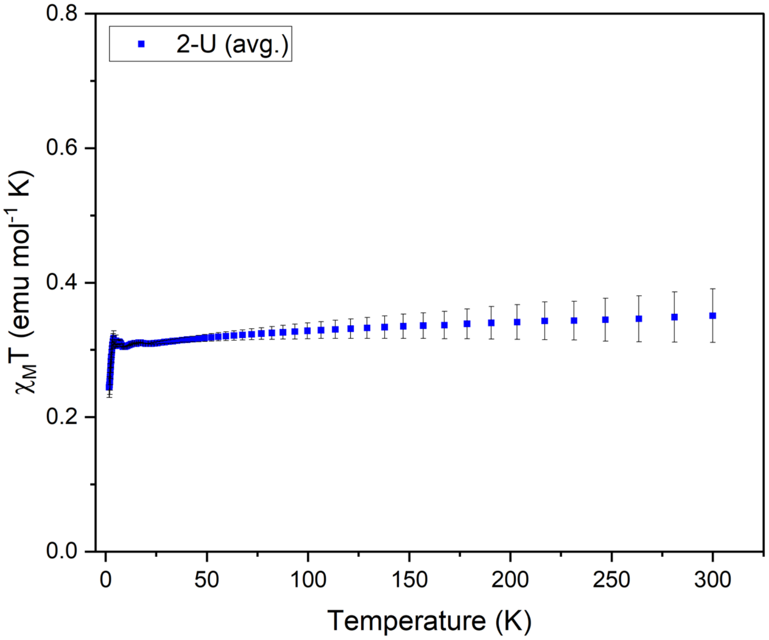

Figure S39. χ_MT vs. temperature for **2-U** under a dc applied field of 1 T. Note: Upon measurement of individually prepared samples of **2-U** from separate batches, some variation was observed. This is attributed to the overall low moment of the sample, in addition to its large molar mass and diamagnetic contribution. The shape and trends observed were consistent across samples, and the data presented are an average of two independent samples, with error bars representing the uncertainty. The χ_MT magnitude and curve shape is consistent with pentavalent uranium,[4] and supports the oxidation state assignment.

Single-Crystal X-ray Diffraction

Table S3. Crystallographic parameters

	**1-U**	**2-U**	**3-U**
Formula	$C_{48}H_{108}N_4P_4U$	$C_{76}H_{118}BF_{20}N_4OP_4U$	$C_{100}H_{118}B_2F_{40}N_4OP_4U$
Formula Weight/gmol^{-1}	1139.36	1856.46	2535.51
Collection Temperature/K	100(2)	100(2)	100(2)
Space Group	$P2_1/n$	P-1	C2/c
System	Monoclinic	Triclinic	Monoclinic
Resolution/Å	0.60	0.71	0.85
a/Å	12.8517(9)	12.4654(6)	14.4582(12)
b/Å	21.7661(14)	16.6746(8)	36.962(3)
c/Å	21.5390(15)	20.3649(9)	22.6993(19)
α/°	90	91.078(2)	90
β/°	94.875(3)	98.373(2)	108.013(3)
γ/°	90	92.680(2)	90
Volume/Å^3	6003.3(7)	4181.9(3)	11536.2(16)
Z[Z']	4[1]	2[1]	4[0.5]
ρ/gcm^{-3}	1.261	1.474	1.460
μ/mm^{-1}	2.843	2.106	1.572
F(000)	2388.0	1894.0	5104.0
Crystal Size/mm^3	0.414x0.364x0.22	0.355x0.207x0.09	0.248x0.124x0.095
Radiation Type	MoKα (λ=0.71073)	MoKα (λ=0.71073)	MoKα (λ=0.71073)
Physical Description	pink prism	red prism	black prism
Collection 2θ range/°	3.69 to 72.634	4.014 to 60.066	3.774 to 49.424
Index Ranges	$-21 \le h \le 21$<br>$-36 \le k \le 36$<br>$-35 \le l \le 31$	$-17 \le h \le 17$<br>$-23 \le k \le 23$<br>$-28 \le l \le 28$	$-17 \le h \le 17$,<br>$-43 \le k \le 43$,<br>$-26 \le l \le 26$
Reflections Collected	335955	140846	104445
Independent Reflections	29091 [R_{int}=0.0911, R_{sigma}=0.0410]	24473 [R_{int}=0.0562, R_{sigma}=0.0406]	9835 [R_{int}= 0.0723, R_{sigma}=0.0334]
Data/Restraints/Parameters	29091/7/594	24473/0/1010	9835/0/705
Goodness of Fit	1.027	1.062	1.057
Final R Indices (I ≥2σ)	R_1=0.0283, wR_2=0.0560	R_1=0.0314, wR_2=0.0679	R_1=0.0311, wR_2=0.0716
Final R Indices (all data)	R_1 = 0.0418, wR_2 = 0.0598	R_1=0.0392, wR_2= 0.0712	R_1=0.0379, wR_2=0.0754
Largest Diff. Peak/Hole/eA^3	0.59/-0.76	1.56/-0.93	1.66/-0.73
Completeness to 2θ/%	99.9	99.9	100
CCDC Number	2384999	2385000	2385001

Experimental and Refinement Details

Crystals suitable for X-ray diffraction were coated in Cargille-NVH oil in a glovebox and transferred to the diffractometer in a capped scintillation vial. Crystals were mounted on a nylon loop on a Bruker D8 VENTURE diffractometer dual wavelength Mo/Cu four-circle diffractometer with a microfocus sealed X-ray tube using a mirror optics as monochromator and a Bruker PHOTON III detector. The diffractometer is equipped with an Oxford Cryostream 800 cryostat and crystals were cooled and kept at T = 100(2) K during data collection. All data were integrated in APEX4[5] with SAINT and a multi-scan absorption correction using SADABS was applied.[6] The structures were solved with the ShelXT structure solution program using the Intrinsic Phasing solution method and refined by full-matrix least-squares methods against F^2 using SHELXL-2014[7] and Olex2 1.5-alpha[8] as the graphical interface. All non-hydrogen atoms were refined with anisotropic displacement parameters. All hydrogen atoms were refined isotropically on calculated positions using a riding model with their Uiso values constrained to 1.5 times the Ueq of their pivot atoms for terminal sp^3 carbon atoms and 1.2 times for all other carbon atoms. Crystallographic data for the structures reported in this paper have been deposited with the Cambridge Crystallographic Data Centre

Solution of **3-U**:

Refinement of the data modeling the [$U^{6+}(NP^tBu_3)_4$] and [$BArF_{20}$]$^-$ moieties with a half equivalent of diethyl ether in the asymmetric unit. A C level CheckCif alert noted an accessible solvent void, and the data was reexamined. Highly disordered diethyl ether in the accessible voids was found, however, it could not be readily modeled. A solvent mask (SQUEEZE)[9] was applied, which accounted for density consistent with five molecules of diethyl ether per unit cell. The dataset was trimmed to 0.85 Å resolution due to poor intensity of high-angle reflections.

Table S4. Bond distance metrics

Atoms	Distance (Å)	Atoms	Distance (Å)	Atoms	Angle (deg)
**1-U**					
U1-N1	2.186(1)	P1-N1	1.555(1)	N3-U1-N4	110.40(6)
U1-N2	2.186(2)	P2-N2	1.554(2)	N4-U1-N1	107.19(6)
U1-N3	2.187(1)	P3-N3	1.560(1)	N1-U1-N3	111.76(6)
U1-N4	2.182(1)	P4-N4	1.556(1)	N3-U1-N2	107.66(6)
				N4-U1-N2	109.30(6)
				N1-U1-N2	110.53(6)
**2-U**					
U1-N1	2.107(2)	P1-N1	1.588(2)	N3-U1-N2	112.26(8)
U1-N2	2.102(2)	P2-N2	1.587(2)	N2-U1-N4	105.99(8)
U1-N3	2.111(2)	P3-N3	1.590(2)	N4-U1-N1	113.03(9)
U1-N4	2.108(2)	P4-N4	1.588(2)	N1-U1-N3	106.74(9)
				N2-U1-N1	109.22(9)
				N3-U1-N4	109.7(8)
**3-U**					
U1-N1	2.055(3)	P1-N1	1.622(3)	N1-U1-N1‘	110.9(2)
U1-N2	2.038(3)	P2-N2	1.628(3)	N2-U1-N1‘	107.8(1)
				N2‘-U1-N1	107.8(1)
				N2-U1-N1‘	110.6(1)
				N2‘-U1-N1‘	110.6(1)
				N2-U1-N2‘	109.3(2)

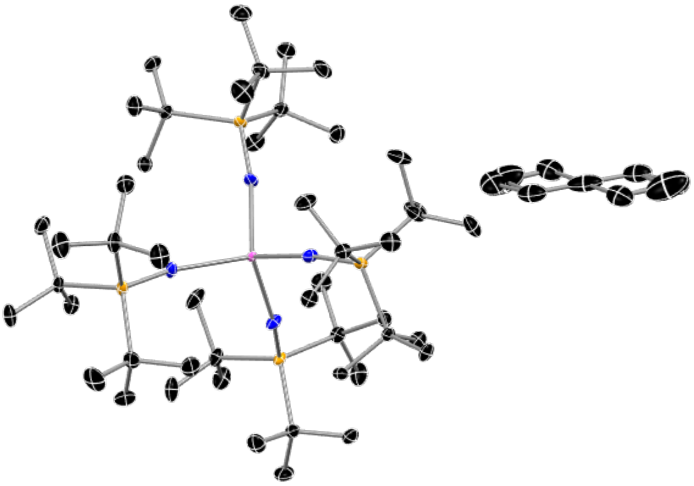

Figure S40. Crystallographic asymmetric unit of **1-U**. pink = U, orange = P, blue = N, black = C. Hydrogen atoms removed for clarity, thermal ellipsoids drawn at 50% probability. *n*-Pentane is disordered over two positions with 50/50 occupancy.

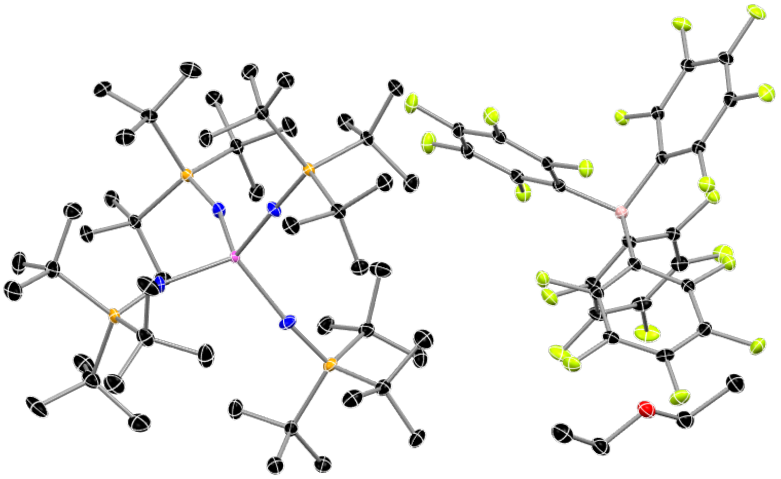

Figure S41. Crystallographic asymmetric unit of **2-U**. pink = U, orange = P, yellow = F, red = O, blue = N, black = C, peach = B. Hydrogen atoms removed for clarity, thermal ellipsoids drawn at 50% probability.

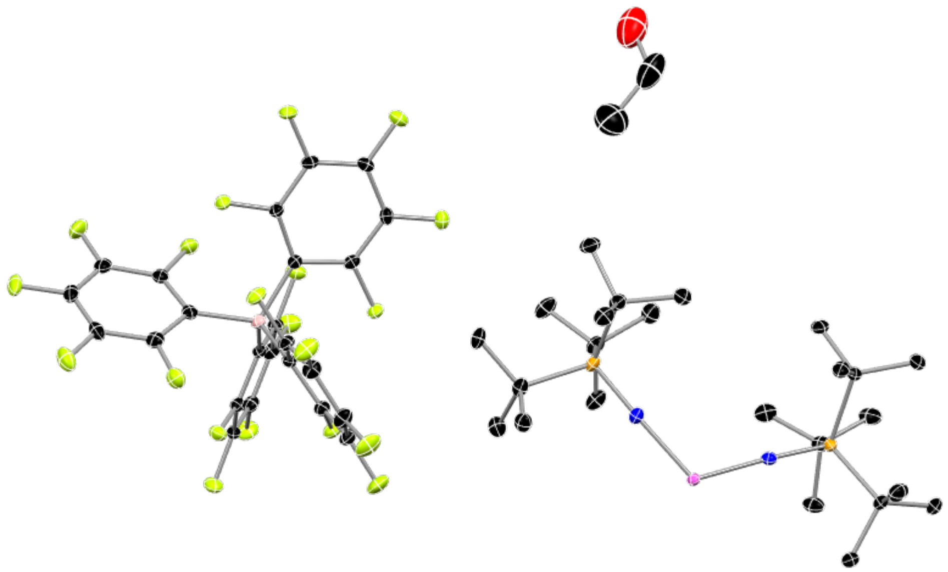

Figure S42. Crystallographic asymmetric unit of **3-U**. pink = U, orange = P, yellow = F, red = O, blue = N, black = C, peach = B. Hydrogen atoms removed for clarity, thermal ellipsoids drawn at 50% probability.

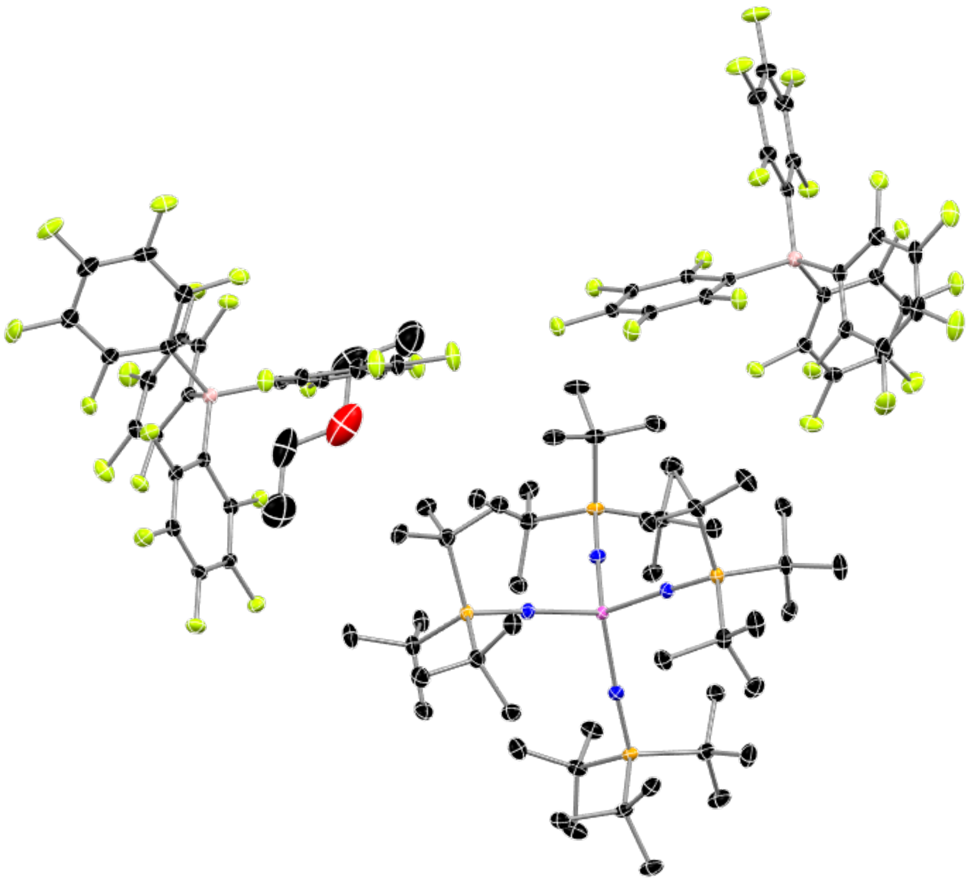

Figure S43. Symmetry-generated structure of **3-U**. pink = U, orange = P, yellow = F, red = O, blue = N, black = C, peach = B. Hydrogen atoms removed for clarity, thermal ellipsoids drawn at 50% probability.

References

1. Tuncuk, A.; Akcil, A., Iron removal in production of purified quartz by hydrometallurgical process. *Int. J. Miner. Process.* **2016,** *153*, 44-50.
2. Boggiano, A. C.; Studvick, C. M.; Steiner, A.; Bacsa, J.; Popov, I. A.; La Pierre, H. S., Structural distortion by alkali metal cations modulates the redox and electronic properties of Ce^{3+} imidophosphorane complexes. *Chem. Sci.* **2023,** *14* (42), 11708-11717.
3. Bain, G. A.; Berry, J. F., Diamagnetic Corrections and Pascal's Constants. *J. Chem. Educ.* **2008,** *85* (4), 532.
4. Kindra, D. R.; Evans, W. J., Magnetic Susceptibility of Uranium Complexes. *Chem. Rev.* **2014,** *114* (18), 8865-8882.
5. *APEX 4*, V8.40B; Bruker: Madison, WI, USA.
6. Krause, L.; Herbst-Irmer, R.; Sheldrick, G. M.; Stalke, D., Comparison of silver and molybdenum microfocus X-ray sources for single-crystal structure determination. *J. Appl. Crystallogr.* **2015,** *48* (1), 3-10.
7. Sheldrick, G., Crystal structure refinement with SHELXL. *Acta Crystallogr. C* **2015,** *71* (1), 3-8.
8. Dolomanov, O. V.; Bourhis, L. J.; Gildea, R. J.; Howard, J. A. K.; Puschmann, H., OLEX2: a complete structure solution, refinement and analysis program. *J. Appl. Crystallogr.* **2009,** *42* (2), 339-341.
9. Spek, A., PLATON SQUEEZE: a tool for the calculation of the disordered solvent contribution to the calculated structure factors. *Acta Crystallogr. C* **2015,** *71* (1), 9-18.
